# The epitope arrangement on flavivirus particles contributes to Mab C10’s extraordinary neutralization breadth across Zika and dengue viruses

**DOI:** 10.1016/j.cell.2021.11.010

**Published:** 2021-12-09

**Authors:** Arvind Sharma, Xiaokang Zhang, Wanwisa Dejnirattisai, Xinghong Dai, Danyang Gong, Wiyada Wongwiwat, Stéphane Duquerroy, Alexander Rouvinski, Marie-Christine Vaney, Pablo Guardado-Calvo, Ahmed Haouz, Patrick England, Ren Sun, Z. Hong Zhou, Juthathip Mongkolsapaya, Gavin R. Screaton, Felix A. Rey

**Affiliations:** 1 Institut Pasteur, Université de Paris, CNRS UMR3569, Unité de Virologie Structurale, 75015 Paris, France; 2 Wellcome Centre for Human Genetics, Nuffield Department of Medicine, University of Oxford, Oxford, UK; 3 Department of Microbiology, Immunology and Molecular Genetics, University of California, Los Angeles, Los Angeles, CA 90095, USA; 4 Department of Molecular and Medical Pharmacology, University of California, Los Angeles, Los Angeles, CA 90095, USA; 5 California NanoSystems Institute, University of California, Los Angeles, Los Angeles, CA 90095, USA; 6 Institut Pasteur, Université de Paris, CNRS UMR 3528, Center for Technological Resources and Research, 75015 Paris, France; 7 Dengue Hemorrhagic Fever Research Unit, Office for Research and Development, Faculty of Medicine, Siriraj Hospital, Mahidol University, Bangkok, Thailand; 8 Division of Medical Sciences, University of Oxford, Oxford, UK; 9 Université Paris-Saclay, Faculté des Sciences, F-91405 Orsay, France; 10 Interdisciplinary Center for Brain Information, the Brain Cognition and Brain Disease Institute, Faculty of Life and Health Sciences, Shenzhen Institute of Advanced Technology, Chinese Academy of Sciences, Shenzhen-Hong Kong Institute of Brain Science-Shenzhen Fundamental Research Institutions, Shenzhen, Guangdong 518055, China

**Keywords:** Flaviviruses, Zika virus, Dengue virus, broadly neutralizing antibodies, vaccine design, cryo-EM, X-ray crystallography

## Abstract

The human monoclonal antibody C10 exhibits extraordinary cross-reactivity, potently neutralizing Zika virus (ZIKV) and the four serotypes of dengue virus (DENV1–DENV4). Here we describe a comparative structure-function analysis of C10 bound to the envelope (E) protein dimers of the five viruses it neutralizes. We demonstrate that the C10 Fab has high affinity for ZIKV and DENV1 but not for DENV2, DENV3, and DENV4. We further show that the C10 interaction with the latter viruses requires an E protein conformational landscape that limits binding to only one of the three independent epitopes per virion. This limited affinity is nevertheless counterbalanced by the particle’s icosahedral organization, which allows two different dimers to be reached by both Fab arms of a C10 immunoglobulin. The epitopes’ geometric distribution thus confers C10 its exceptional neutralization breadth. Our results highlight the importance not only of paratope/epitope complementarity but also the topological distribution for epitope-focused vaccine design.

## Highlights

• The C10 Fab’s orientation on E dimers allows bivalent IgG binding to each virion raft
• C10 binding to DENV2 induces E dimer rearrangement by hitting a spring-loaded segment
• Only E dimers with asymmetric environment on DENV2 virions can bind C10
• Bivalent binding to two different E dimers expands C10 neutralization breadth

## Graphical abstract

**Figure:**
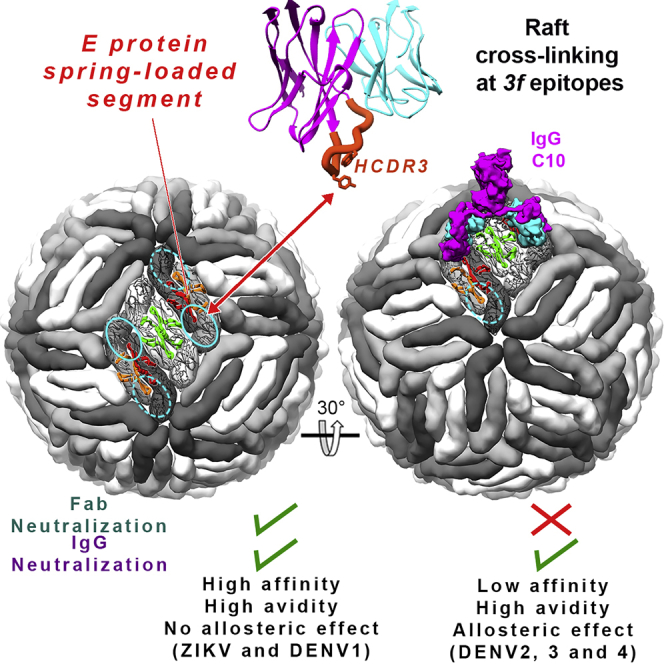

## Teaser

Comparative structure-function analyses reveal how the topological distribution of epitopes explains the cross-reactivity and neutralization of Zika and dengue viruses by the human monoclonal antibody C10.

## Introduction

Flaviviruses are the most important arthropod-borne viral pathogens for humans, causing severe disease around the world (Collins and Metz, 2017). Among them is the highly teratogenic and neurotropic Zika virus (ZIKV), which re-emerged recently (Pierson and Diamond, 2018), and the worldwide distributed dengue viruses of serotype 1-4 (DENV1–DENV4), which impose a very high toll on public health, with 50–100 million cases yearly. The four DENVs cause ∼500,000 hospitalizations annually (Bhatt et al., 2013) of individuals with a hemorrhagic syndrome resulting from vascular leakage (Halstead, 2007). The neutralizing antibodies induced during a DENV or ZIKV infection target the envelope (E) protein (Fibriansah and Lok, 2016) and, with a few exceptions, are serotype specific. Cross-reactive antibodies are also elicited, which, in general, are poorly neutralizing and have been linked to antibody-dependent enhancement (ADE) of the disease upon ulterior heterotypic infection (Halstead, 2014). As a result, no efficient anti-dengue vaccine is currently available (Halstead et al., 2020), and a potential effect of ZIKV vaccination of potentiating a subsequent dengue infection is a concern (Priyamvada et al., 2017). An ideal vaccine should therefore protect simultaneously against all four DENVs as well as ZIKV.

**Figure 1:**
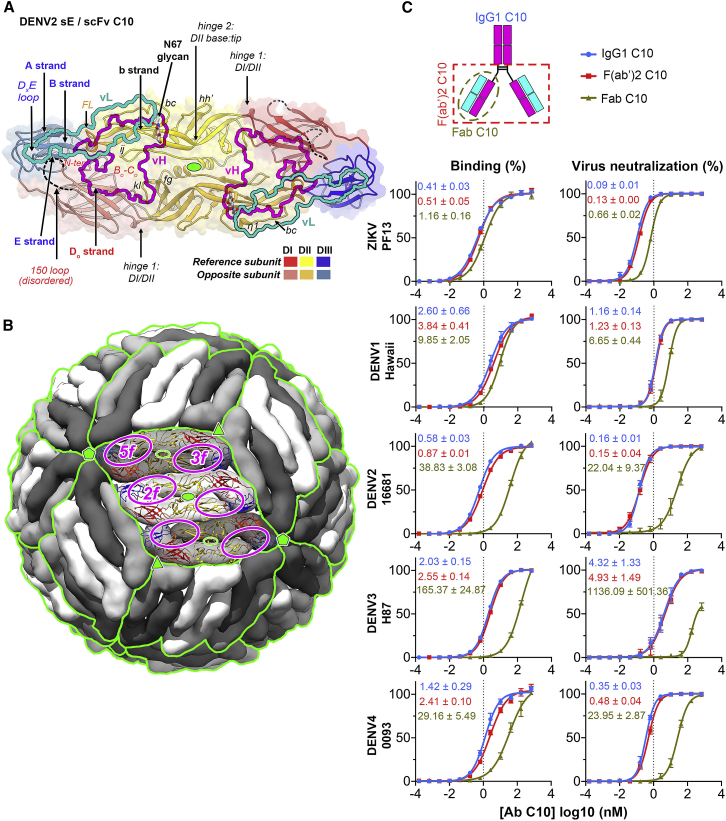
Virus-dependent bivalent versus monovalent C10 neutralization differences (A) The DENV2 sE dimer color-coded by domain with the C10 footprint outlined (heavy chain in magenta, light chain in cyan) (PDB: 4UT9). A green oval at the center marks the molecular 2-fold axis. The sE protomer contributing the fusion loop (FL; orange) or domain III (blue) to the epitope are dubbed “reference” or “opposite” subunit, respectively. (B) The flavivirus mature virion displayed in surface representation with selected icosahedral symmetry axes shown as full green symbols: 2-fold (I2, oval), 3-fold (I3, triangle), and 5-fold (I5, pentagon). 90 E dimers are arranged as 30 “rafts” (green outlines) made of three E dimers. The central, I2 dimer (white) is flanked by two L2 dimers, (light/dark gray) formed about local 2-fold axes (open green ovals). The location of the C10 epitopes is outlined in magenta in the front raft, labeled *2f*, *3f*, and *5f*. (C) Bivalent versus monovalent C10 binding and neutralization. Top: cartoon of IgG1, F(ab’)2, and Fab molecules. Bottom: ELISA titration curves and the estimated apparent dissociation constant (K_D_) (left panels) and neutralization curves with the 50% focus reduction neutralization titer (FRNT 50%) measured on Vero cells (right panels) for ZIKV and DENV1–DENV4 grown in insect cells. Data are from three independent experiments.

Only the members of a special class of human broadly neutralizing antibodies targeting the so-called E dimer epitope (EDE) have been shown to potently neutralize ZIKV and the four DENV serotypes (Barba-Spaeth et al., 2016; Dejnirattisai et al., 2015; Rouvinski et al., 2015). C10, whose *in vivo* protective effect has been demonstrated (Swanstrom et al., 2016), and C8 are among the broadest neutralizing monoclonal antibodies (mAbs) targeting the EDE. Their footprints on the DENV2 and ZIKV E dimer have been structurally defined (Barba-Spaeth et al., 2016; Rouvinski et al., 2015; Zhang et al., 2016; Figure 1A). The epitopes are distributed evenly at the surface of the icosahedral virion, which is composed of 90 E dimers organized in 30 “rafts” of three parallel E dimers (Figure 1B; Kuhn et al., 2002). The 15 icosahedral 2-fold (I2) axes of the particle intersect two diametrically opposed rafts. The molecular 2-fold symmetry axis of the central E dimer (termed “I2 dimer”) in each raft is coincident with an I2 axis, and the flanking E dimers are related only by a local molecular 2-fold axis (L2 dimers) (Figure 1B). One end of the L2 dimer makes inter-raft contacts about the 5-fold (I5) and the other about the 3-fold (I3) icosahedral axes. The environments of the two EDEs of the L2 dimers, termed “*5f*” and “*3f*,” are therefore non-equivalent, whereas they are equivalent (termed “*2f*”) on the I2 dimers (Figure 1B, magenta circles).

Here we explored the structural features that confer antibodies targeting the EDE to such an exceptional breadth. We found that antibody binding geometry is as important as core epitope conservation in conferring broad cross-neutralization. Indeed, despite the relatively even cross-neutralization of bivalent C10 immunoglobulin G1 (IgG1) for ZIKV and the four DENV serotypes, the monovalent C10 antigen binding fragment (Fab) retains neutralization potency only for ZIKV and DENV1. We also show that the C10 paratope is robust to amino acid changes with respect to binding and neutralization potency against ZIKV and DENV1 but not for the other three viruses. In addition, the X-ray structures of C10 in complex with sE from the five viruses it neutralizes showed that, contrary to ZIKV and DENV1, C10 binding stabilized an asymmetric conformation of DENV2, DENV3, and DENV4 sE dimers, with only one of the two epitopes in a conformation compatible with the interactions of E on virions. Cryoelectron microscopy (cryo-EM) studies showed that a C10 single-chain variable fragment (scFv) could bind only one of the two epitopes on the L2 dimer on the DENV2 virion, either *3f* or *5f*, and did not bind the I2 dimers. Furthermore, the X-ray structure of ZIKV sE in complex with a bivalent immunoglobulin hinted at its mode of bivalent binding to virions. The accompanying paper by Lim et al. (2021) in this issue of *Cell* confirms the bivalent binding mode to the two *3f* epitopes per raft and further reveals that C10 binding to the DENV2 virion causes increased hydrogen-deuterium exchange of E protein peptides spanning the epitope, in line with sE dimer distortion revealed by the X-ray structures. Our results show that the gained avidity acquired by bivalent binding largely compensates for the lower affinity of the monovalent Fab arms to the DENV2 E dimer (and, by extrapolation, to DENV3 and DENV4, which display the same epitope organization) as well as for the limited accessibility to only one of the epitopes on L2 dimers.

## Results

### Different monovalent versus bivalent C10 neutralization patterns depending on the virus

We carried out *in vitro* binding and neutralization experiments in parallel using C10 intact bivalent IgG1, bivalent F(ab’)_2_, and monovalent Fab (Figure 1C). The concentration of bivalent C10 required for 50% reduction of infection (IC_50_) of ZIKV and DENV1–DENV4 was in the range of 0.1–4 nM. The monovalent Fab IC_50_ remained in this range for ZIKV and DENV1 but not for DENV2, DENV3, and DENV4, for which it was nearly two logs higher (Figure 1C). The differences in neutralization potency correlated with a significant increase in avidity of the bivalent binders for the latter three viruses. Indeed, the measured C10 IgG half-maximal effective concentration (EC_50_) for virion binding was under a factor of 3 for bivalent versus monovalent binders in the case of ZIKV and DENV1, but it was 10 times higher for DENV2 and DENV4 and almost two logs for DENV3. In contrast, the IC_50_ of bivalent C10 for ZIKV, DENV2, and DENV4 was below 0.5 nM, whereas it was 1 nM and 5 nM for DENV1 and DENV3, respectively. In some of the experiments reported in Figure 1C, the curves for Fab C10 do not reach a plateau, and the inferred EC_50_s and IC_50_s are not reliable. However, because the experiments were all done in parallel on the same cells, they clearly show that the monovalent Fab curves are considerably shifted to the right, whereas this shift is less pronounced in the case of ZIKV and DENV1. A high avidity of the bivalent binders therefore appears to be responsible for the low IC_50_ of C10 for DENV2, DENV3, and DENV4.

### Differential sensitivity to C10 paratope changes

**Figure S1:**
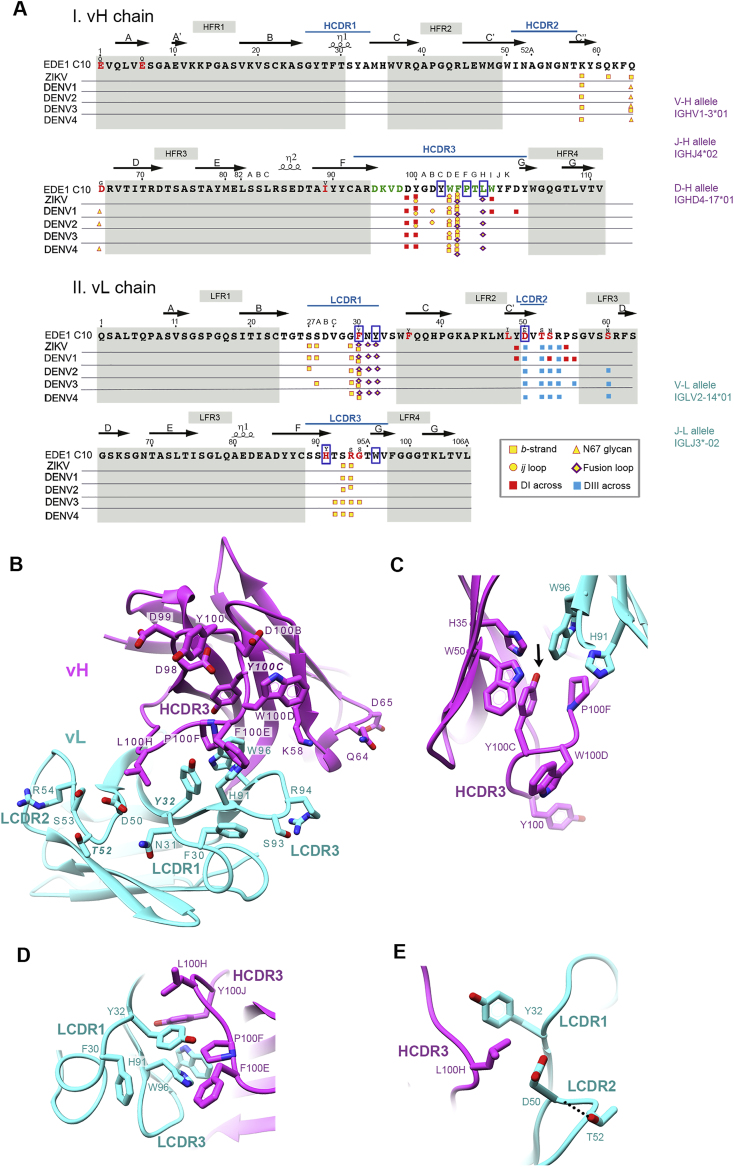
Sequence analysis of C10 and interacting residues of the paratope, related to Figure 2 (A) Amino acid sequence of the C10 heavy (i) and light chains (ii) numbered according to the Kabat convention with the CDRs in white background. The CDRs in the IGMT convention are indicated by a blue line over the sequence. Somatic mutations are marked with red fonts, with the germline aa indicated immediately above. Residues arising from nucleotide insertions at the recombination sites (“N” residues) are in green. The five rows below the sequences mark antigen contacting residues as observed in the corresponding X-ray structures of sE in complex with C10 (see colored key at the bottom right inset). Boxed are key residues at the interface of heavy and light chains important for maintaining the conformational integrity of the paratope. The germline alleles are quoted on the right. (B) The C10 paratope as observed in the X-ray structure of the complex with ZIKV sE complex at 2.1 Å resolution viewed from the antigen and with important residues labeled (magenta, heavy chain; cyan, light chain). (C) HCDR3 residue Y^H100C^ (black arrow) and its interactions at the V_H_/V_L_ interface to allow the correct exposure of paratope residues at the tip of the HCDR3. This structural role is reflected in the strong phenotype of the Y^H100C^ mutant. (D) LCDR1 residue Y^L32^ and its structuring role at the heavy/light chain interface. (E) The LCDR2 T^L52^ side chain makes a bond with the backbone carbonyl group of D^L50^, which in turn packs against residue L^H100H^ of the HCDR3. The strong effect of the T^L52^A mutation - like that of Y^L32^A (Figure 2B), likely reflects conformational disruption of both, HCDR3 and LCDR1 loops.

**Figure 2:**
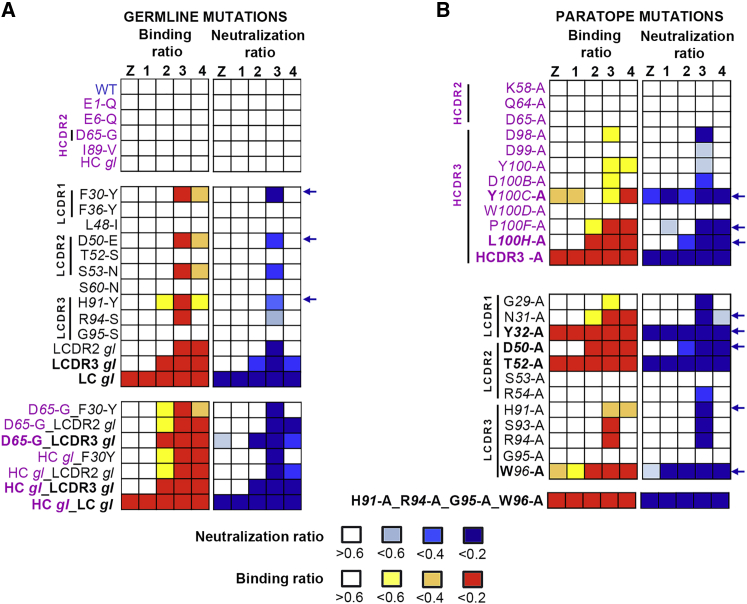
Robustness of the C10 paratope to point mutations Binding and neutralization ratios of EDE1 C10 mutants are color-coded as the ratio to wild-type according to the key. The C10 residues mutated are presented in Kabat numbering, with light and heavy chains marked with black and magenta font, respectively, highlighting in bold those that most severely affect neutralization breadth. Blue arrows indicate heavy/light-chain interface residues. (A) Germline reversion mutants (see also Figure S1A). (B) Alanine scanning of the paratope. See also Table S1.

C10 underwent modest somatic hypermutation: the amino acid (aa) sequence identities of the heavy and light chain with the corresponding inferred germlines are 95.9% and 89.8%, respectively (Figure S1A). None of the reversions to the V_H_ gene sequence significantly affected binding or neutralization, neither individually nor in combination (Figure 2A, top panel; Table S1). In contrast, five of 10 individual reversions in the light chain did reduce neutralization breadth (Figure 2A, center panel). Only the combined reversion to germline of all somatic mutations in the light chain knocked down binding to all five viruses.

We further alanine-scanned C10 in or near the paratope (Figure S1B), as informed by the structure of the DENV2 sE/C10 complex (Rouvinski et al., 2015). The single mutations that knocked out neutralization of all five virus were Y^H100C^A, Y^L32^A, and T^L52^A. The side chains of these three residues are important for organizing the paratope (Figures S1C–S1E). Of the heavy-chain residues that contact the epitope, mutations P^H100F^A and L^H100H^A affected neutralization breadth without significantly altering ZIKV or DENV1 neutralization. Only turning the whole HCDR3 into a polyalanine stretch knocked out binding to all five viruses, highlighting the robustness of the paratope to point mutations, with stronger effects in neutralization of DENV3 and DENV4.

### X-ray structures of the sE/C10 complexes of ZIKV, DENV1, DENV3, and DENV4

**Figure S2:**
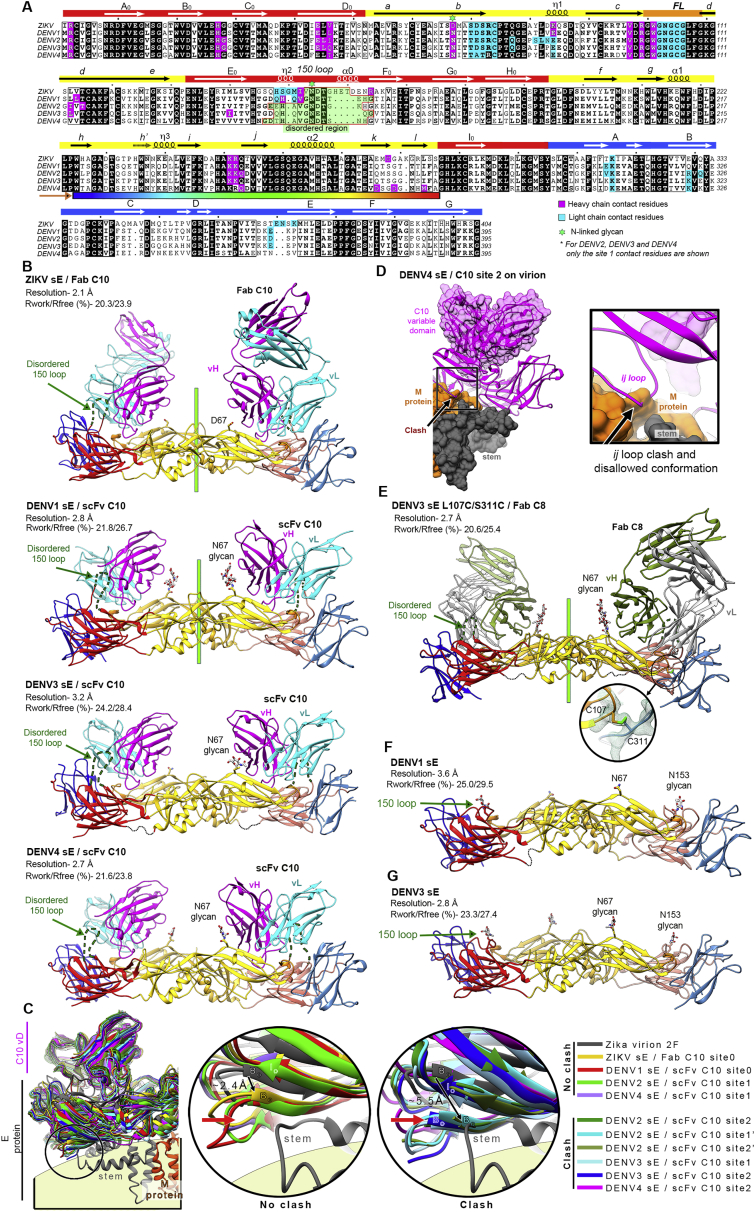
Amino-acid sequence alignment of sE from ZIKV and DENV1-DENV4 and their X-ray structures in complex with C10, related to Figure 3 (A) Amino acid sequence alignment of sE of ZIKV and DENV1-4. A black background highlights strict aa conservation. Residues contacted by C10 in each of the complexes are indicated in a magenta (heavy chain) or cyan (light chain) background. The domain organization (domain1 red, domain II yellow, fusion peptide orange and domain III in blue) and the secondary structure elements are indicated together with their labels above the sequences. A bar ramp-colored from blue to red indicates the segment highlighted in Figure 6A. (B) X-ray structures of ZIKV sE in complex with C10 Fab and of DENV1, DENV3 and DENV4 in complex with C10 scFv. The resolution and crystallographic statistic are quoted, and are provided in detail in Table S2. The 2-fold molecular symmetry of the ZIKV and DENV1 complexes, coincident with a 2-fold crystallographic axis, is drawn in green. The DENV4 and DENV3 complex were not 2-fold symmetric, and displayed significant variation at the antibody/antigen interface. (C) Some sE conformations observed in the complexes with C10 are incompatible with its interactions on virions. The left panel shows an alignment of the X-ray structures on the domain II tip of the E protein on the ZIKV virion at 3.1Å resolution (PDB: 6CO8). The view corresponds to Figure 3A rotated by 180 degrees about a vertical axis. The circle marks the location of the inset shown in the right panels, color-coded according to the rightmost panel. (D) X-ray structure of the DENV4 (sE/C10)_2_ dimer site 2 (in magenta) aligned on the domain II tip of the Zika virion cryo-EM structure (6CO8), showing that the *ij* hairpin conformation in the crystal would clash with protein M (in orange), as highlighted in the right inset (black arrow). The E stem (the region C-terminal to domain III that connects to the trans-membrane anchor, and absent in sE) is displayed in gray. (E) X-ray structure of DENV3 sE L107C/ S311C double cysteine mutant (Rouvinski et al., 2017) bound to Fab C8. The crystallographic 2-fold axis is shown in green. The inset shows the engineered disulfide and 2FoFc map density at 1.2 σ. (F-G) X-ray structures of unbound DENV1 sE and DENV3 sE (see Table S2).

**Figure 3:**
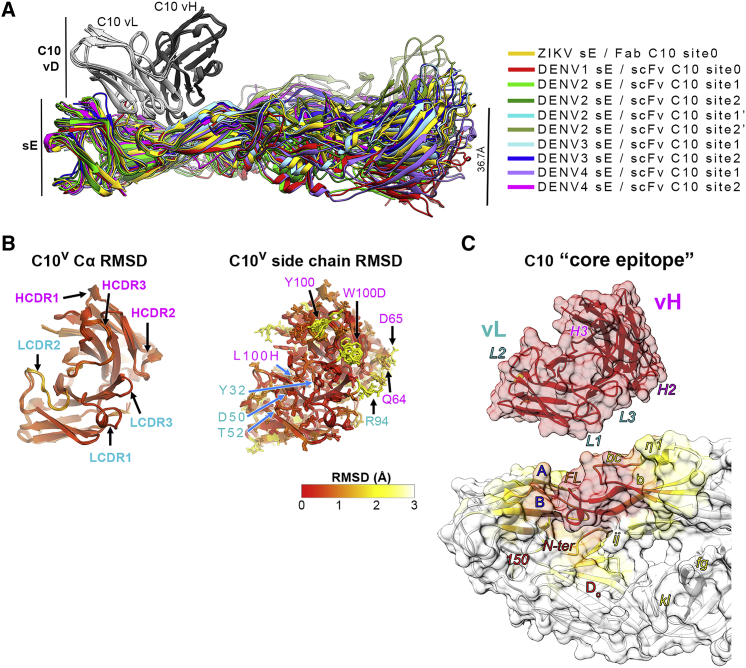
X-ray structures of C10 bound to ZIKV and DENV1–DENV4 sE (A) The ten snapshots of C10 (gray) bound to sE (color-coded as in the key) superposed on the C10^V^ Cα atoms. The second antibody bound per sE dimer is not displayed for clarity (see also Figure S2). (B) The RMSD of the C10^V^ main-chain atoms calculated upon the alignment shown in (A), color-coded on the backbone ribbon (left panel) and on all atoms shown as sticks (right panel) according to the color bar key. Black or blue arrows point to paratope residues displaying high or low side-chain rotamer variability, respectively. Light- and heavy-chain residues are labeled in cyan and magenta, respectively. (C) The C10 core epitope and open book representation of the ZIKV/C10 complex. The mean RMSD of backbone atoms calculated upon the superposition shown in (A) is color coded on a semi-transparent surface. The C10 CDRs and selected sE elements are labeled.

We used C10 Fab and scFv to obtain crystals in complex with sE from ZIKV, DENV1, DENV3, and DENV4 (see the sE aa sequence alignment in Figure S2A). We determined the corresponding X-ray structures to 2.1-, 2.8-, 3.2-, and 2.7-Å resolution, respectively (Figure S2B; Table S2) by molecular replacement. The crystals of ZIKV and DENV1 had one sE/C10 protomer in the asymmetric unit (AU), and a crystallographic 2-fold axis generated a symmetric sE dimer bound to two antibody fragments: Fab and scFv, respectively. The DENV3 and DENV4 crystals instead contained one sE dimer with two scFv C10 bound in the AU, with considerable differences at the two antibody-antigen interfaces within the dimer (Table S3). As discussed below, these data allowed comparative structural analysis of a total of 10 independent snapshots, each consisting of half an (sE/C10^V^)_2_ dimer, where C10^V^ denotes the antibody variable portion made of domains V_H_ and V_L_ of the heavy and light chains, irrespective of the presence of Fab or scFv in the crystals. These half-dimer snapshots are as follows: one each for the ZIKV and DENV1 complexes, two each for DENV3 and DENV4, and four for the previously reported structure of the DENV2 complex (PDB: 4UT9), which featured two (sE/scFv)_2_ dimers in the crystal AU (Rouvinski et al., 2015). The loops from C10 complementarity determining regions (CDRs) in interaction with sE in each of these snapshots are displayed in Figures S4A–S4F. Figure 3A shows an alignment of these 10 snapshots on the C10 backbone.

The C10^V^ backbone remained essentially the same in all complexes (Figures 3A and 3B), although some bulky side chains of the paratope exhibited significantly different rotamers (Figure 3B). This variability in side-chain conformation is in line with the observed robustness of the C10 paratope presented in Figure 2: mutation of residues with low side-chain root-mean-square deviation (RMSD) affected binding and neutralization breadth whereas mutation of residues with variable rotamers did not. Alignment on the C10^V^ backbone also brought into alignment the epitope region, where the RMSD between Cα atoms of the epitope increased with distance to the paratope (Figure 3C). We defined the C10 epitope “core” as the E protein regions in which the RMSDs remained similar to that of the C10^V^ Cα atoms used in the alignment. As expected, the core epitope included the region of the *b* strand and the fusion loop at the tip of domain II of the reference subunit (colored red in Figure 3C). The epitope periphery, shown in yellow in Figure 3C, includes the *ij* hairpin, which is also part of the domain II tip (Figure 1A) and is mobile in the structures, as well as the E protein atoms of the opposite subunit. Residues in the epitope periphery adjust differently in each complex to conform to the paratope.

Structural alignment of the X-ray snapshots above onto the core epitope of E in the available cryo-EM structures of the ZIKV virion at 3.1-Å resolution (Sevvana et al., 2018) and of the DENV2 virion at 2.6-Å resolution (Hardy et al., 2021) showed that the symmetric ZIKV and DENV1 (sE/C10^V^)_2_ half-dimers well matched the conformation of E on the virion. In contrast, only one of the two half-dimers of the asymmetric complexes was compatible with the conformation of E on virions (Figures S2C and S2D). The epitope periphery of the second binding site showed clashes in each case with the underlying M protein or the E stem. For the purpose of our descriptions below, we termed the two half-dimer snapshots per asymmetric dimer sites 1 and 2 to mean the compatible and non-compatible epitope with the interactions on the virion, respectively.

### Cryo-EM structure of C10 scFv bound to the DENV2 virion

**Figure 4:**
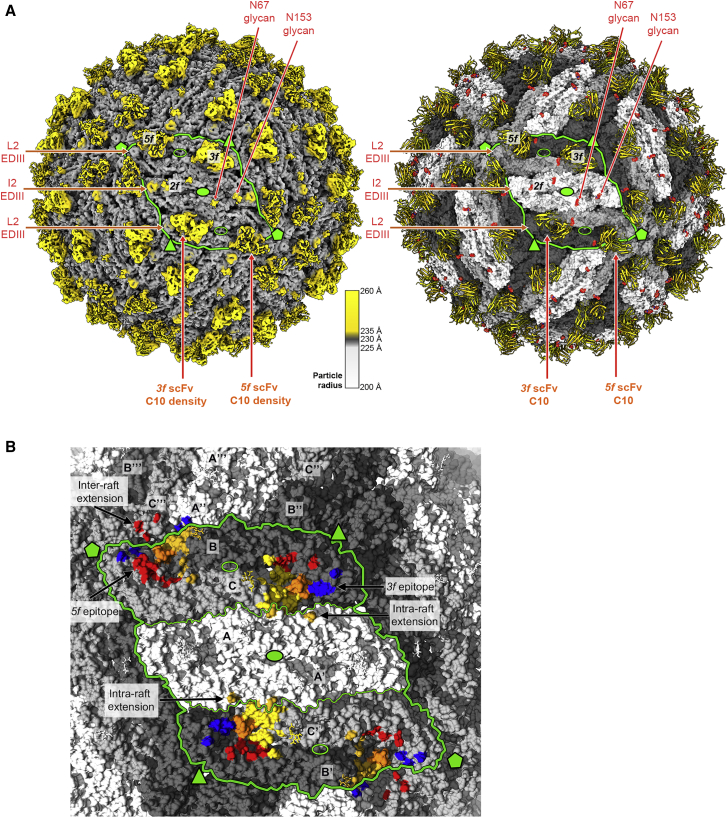
Cryo-EM structure of the DENV2 virion bound to scFv (A) Cryo-EM density map colored according to particle radius as in the key, adjusted to highlight in yellow densities projecting beyond a radius of ∼235 Å, which encompasses most of the E protein layer (gray). The projections at higher radii correspond to the scFvs together with the Asn67-linked glycan on the L2 dimers and to domain III and the Asn67- and Asn153-linked glycans on the I2 dimer. Domain III projects out farther in the I2 than in the L2 dimers. The epitopes are labeled as in Figure 1B, and a similar green outline highlights one raft. The right panel shows the model built into the cryo-EM density of the left panel, with the E protein colored as in Figure 1B and shown in surface representation, with the attached glycans displayed as red spheres. The bound scFv at the *3f* and *5f* epitopes is shown as yellow ribbons. (B) C10 footprint on the virion. The E subunits are colored white/gray as in Figure 1B, with the C10 footprint colored by E domains as in Figure 1A. Within the outlined raft, three independent E polypeptides are labeled A, B, and C, and the I2 related counterparts are labeled A’, B’, and C’, with I2 dimers made by subunit AA’ and L2 by BC and B’C’. The top left I5 axis relates subunits A, B, and C to A’’’, B’’’, and C’’’ and A’, B’, and C’ to A’’, B’’, and C’’ of the adjacent raft. The C10 footprint on the contacting L2 dimer includes inter-raft contacts to domain I of C’’’ and domain III of A’’ at the *5f* epitope and to domain II of A at the *3f* site.

**Figure S3:**
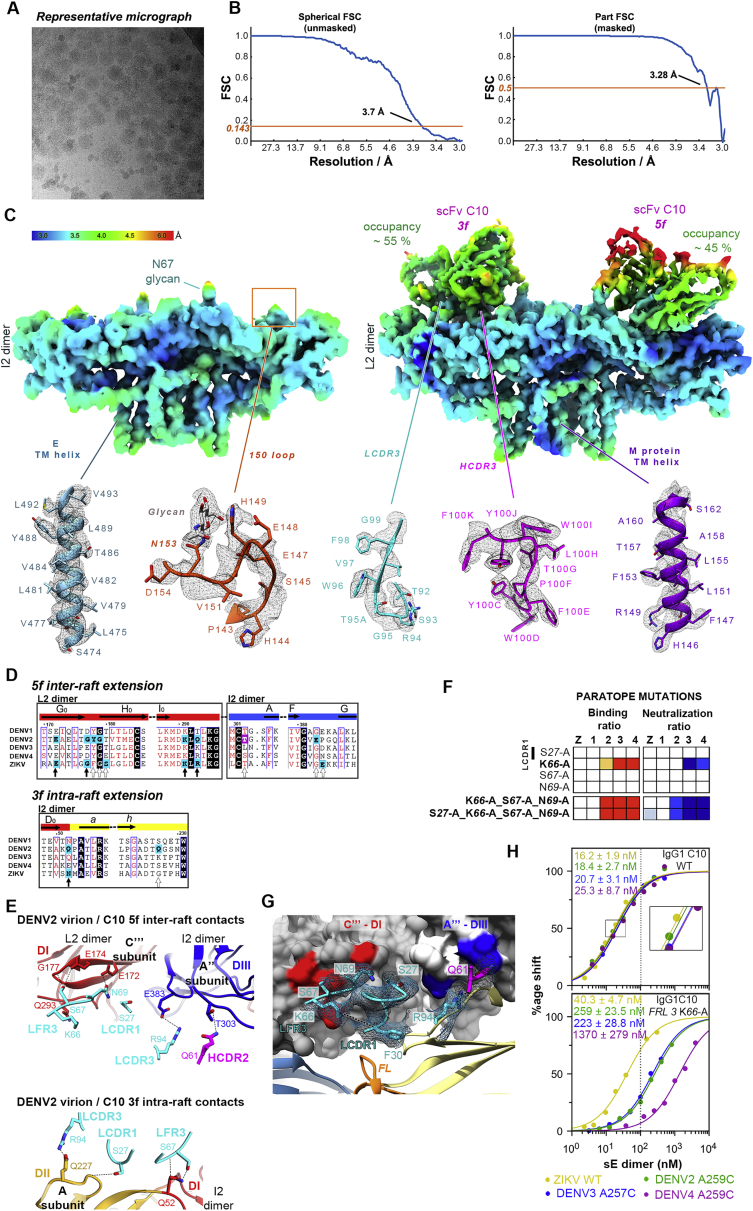
The cryo-EM structure of the DENV2 virion bound to C10, related to Figure 4 (A) Representative cryo-micrograph showing a field of DENV2 virions in complex with C10 scFv, from which the cryo-EM map was derived. (B) Fourier shell correlation function indicating an overall resolution of about 3.7 Å. Which was extended to 3.3Å upon using a mask (see Stat Methods). (C) Top panels: the final cryo-EM density around the I2 (left) and L2 (right) dimer colored according to local resolution as estimated by ResMap (Kucukelbir et al., 2014). Bottom panels: representative densities of the final model within cryo-EM density. From left to right: C-terminal TM helix in E; 150 loop tranced in the I2 dimer; LCDR3 as traced in the scFv bound to the *3f* site; HCDR3 traced at the same site; and C-terminal TM helix of M (D) Portion of the aa sequence alignment of the E protein showing the conservation of the residues contacted at the epitope extensions on the virion. Full vertical arrows below the alignment indicate residues observed in contact with C10 in both in the ZIKV and DENV2 cryo-EM structures. Empty arrows mark residues contacted by C10 either in ZIKV or DENV2 virion. Residues forming epitope extensions are highlighted in a magenta (heavy chain) or cyan (light chain) background. (E) C10 contacts with the adjacent raft at the *5f* (top panel) and at the *3f* (bottom panel) epitope in the DENV2 virion. Only the L2 and I2 dimers of the adjacent raft are displayed as ribbons, and the C10 loops making contacts are shown as sticks color coded by atom type with carbon atoms cyan and magenta for light and heavy chain, respectively. The residues making lateral contacts were identified using a distance cutoff of 5 Å. (F) Alanine scanning of C10 residues contacting epitope extensions on the virion. The binding and neutralization ratios for C10 mutants bearing alanine at the indicated positions is displayed as in Figure 2 (related to Table S1). (G) Close-up of the C10 contact with the adjacent raft (labeled C’’’-DI and A’’’-DIII, see Figure 4B) at the *5f* site. The E dimers of the adjacent raft are shown in surface representation, and the E dimer on which C10 is bound is shown as ribbons colored coded by domains as in Figure 1A. For clarity, only the C10 loops in contact with the adjacent dimers are shown, in cyan and magenta for light and heavy chain, respectively. The cryo-EM density for the displayed C10 residues is represented in a cyan mesh. The dotted black line shows a polar bond of the K^L66^ side chain with the backbone of LCDR1 residue F^L30^. (H) Steady state binding of WT IgG1 C10 and the K^L66^A mutants to the recombinant sE dimers and the measured *K*_*D*_ values. The color code is indicated below the Figure. The C10 K^L66^A mutant also affected binding to the isolated sE dimers, indicating that the effect of this mutation is due to its role in structuring the paratope and not because of the inter-raft contact.

**Figure S4:**
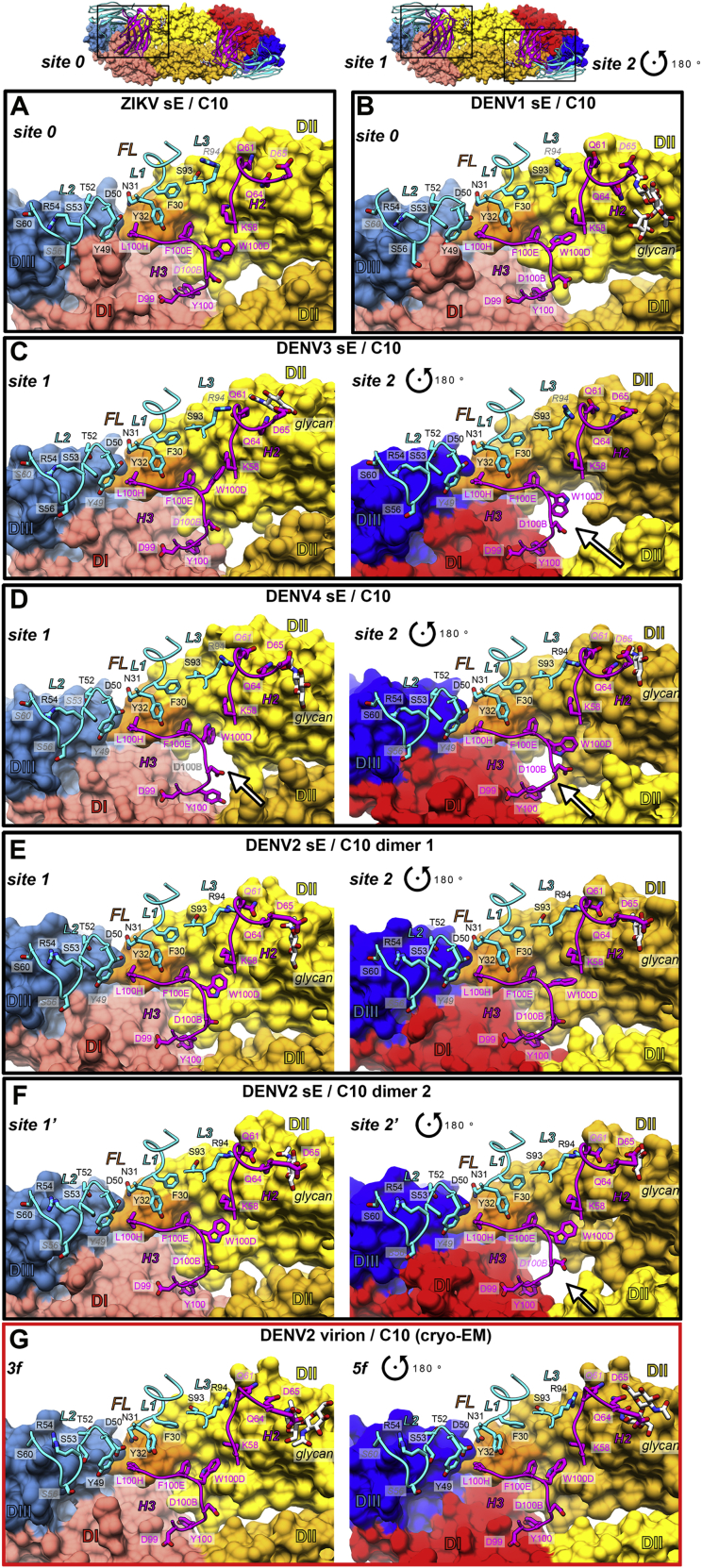
Interactions of the C10 CDRs with E in the various complexes analyzed, related to Figure 5 The two insets in the top row display two representative complexes displayed below: a fully symmetric (sE/C10)_2_ dimer with two identical C10 binding sites (left), and an asymmetrical (sE/C10)_2_ dimer with two different binding sites. Accordingly, only one site (site 0, symmetric dimers) and both sites (sites 1 and 2, asymmetric dimers) are enlarged in the panels below. For ease of comparison, the site 2 is shown in the closeup after a 180° rotation about the vertical axes (as indicated by the symbol). The coloring of the E domains in the two protomers is also used as a guide: bright and washed colors match those in the inset. The C10 variable region is shown as ribbons with the heavy and light chains colored magenta and cyan, respectively. The structures shown in all the other panels were aligned on C10^V^ and, except for the two insets, only the C10 CDRs are shown for clarity. The paratope residues making contact are shown as sticks. Paratope residues that make contact in some structures, but not in the panel being displayed, are labeled in fainted font color, for instance S^L56^ or D^H100B^ in panel A. The various panels show that the HCDR3 protrusion adapts differently to the various complexes, and often induces disorder in the *ij* and *kl* hairpin region in the asymmetric complexes (white arrows), and affect the sE dimer differently in both binding sites in the sE dimer. The most variable is the conformation of the W^H100D^, at the very tip of the HCDR3.

To understand the binding of C10 in the context of the virus particle, we determined the cryo-EM structure of the DENV2 virion in complex with C10 scFv (Figures 4A and S3A). We obtained a cryo-EM map to ∼3.3-Å overall resolution (Figure S3B), with the M/E glycoprotein shell well resolved. We found interpretable density for the scFv bound to the *5f* and *3f* epitopes (Figure S3C) but not at the *2f* site. Instead, the cryo-EM density for the 150 loop of E at the *2f* site was clear, and we could build it readily (Figure S3C). C10 binding has been shown in previous structural studies to induce disorder in the 150 loop (Rouvinski et al., 2015), as confirmed here in the X-ray structures reported (Figure S2B). This was indeed the case for the L2 dimer, where we found no density for the 150 loop and could build the C10 scFv instead. Despite a resolution gradient that resulted in poorer definition for the distal end of the scFv, most of the side chains in the paratope were well resolved, as were those in E and M in the virion (Figure S3, Figure S4C and S4G). Comparison of the level of the cryo-EM density of the CDRs bound to the *3f* and *5f* sites showed that it was about 50% that of the E protein, indicating that each binding site had only half occupancy despite a stoichiometric excess of C10 scFv over E protein when preparing the complex for cryo-EM (STAR Methods). In contrast, the previously reported cryo-EM reconstruction of the ZIKV virion in complex with Fab C10 determined to ∼4-Å resolution (Zhang et al., 2016) showed even coverage of the particle, with the Fabs bound at all three non-equivalent epitopes (*2f*, *3f*, and *5f*, labeled in Figure 1B), which is in line with the symmetric, undistorted sE dimer observed here in the X-ray structure of the ZIKV sE/C10 complex.

### C10 epitope extensions at the particle surface

The DENV2/C10 scFv cryo-EM map further showed that, as in the complex with ZIKV, the antigen/antibody contact region extended into the adjacent dimers at the particle surface, with the footprint at the *3f* epitope augmented by contacts of the C10 light chain with E domain II of the I2 dimer in the raft (intra-raft extension), whereas the footprint at the *5f* epitope also showed contacts with domains I and III of the L2 and I2 dimers, respectively, of the anti-clockwise 5-fold related raft (inter-raft extension) (Figure 4B; see also Figure 1B). The same residues of the C10 light-chain framework region 3 (LFR3) are responsible for the interactions in both extensions, but the antigen residues contacted in each case are not chemically similar (Figures S3D and S3E). The lateral contacts of the C10 LFR3 thus appear to be only accessory and are not binding determinants, as demonstrated by mutagenesis of the C10-interacting residues (Figure S3F). Only mutation of K^L66^, which also positions the LCDR1 for recognition of the fusion loop in the epitope core (Figure S3G), entailed a reduction in neutralization breadth (Figure S3F; Table S1). Biolayer interferometry (BLI) experiments using recombinant DENV2, DENV3, and DENV4 sE dimers further confirmed that the affinity of the K^L66^A C10 mAb mutant dropped by more than 1 log, whereas it was affected only by a factor of 2 for ZIKV (Figure S3H). Because the sE dimers in solution are not expected to follow the raft arrangement of mature virions, these results demonstrate that, although the observed contacts to adjacent dimers may help C10 lock the E protein in the rafts in its pre-fusion conformation, as suggested by Zhang et al. (2016), the contacts at these epitope extensions are not binding determinants and do not contribute to the C10 neutralization breadth.

### C10 docking axes

**Figure 5:**
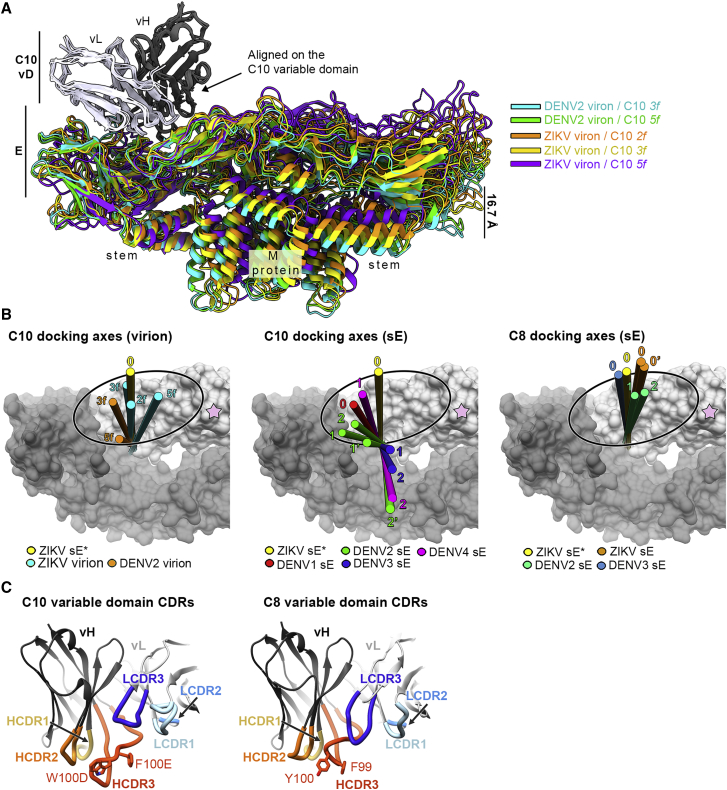
C10 docking on virions and comparison with soluble sE dimers (A) The five cryo-EM C10/E snapshots displayed upon alignment on C10^V^ bound to the *2f*, *3f*, and *5f* epitopes on the ZIKV virion (PDB: 5H37) and to the *3f* and *5f* epitopes of the DENV2 virion (i.e., equivalent to Figure 3A with the 10 X-ray C10/sE snapshots) (Table S4). (B) C10 docking axes (defined as the axis of the transformation relating the core β sandwiches of the C10 V^H^ and V^L^ domains). Instead of aligning on the antibody, each structural snapshot was aligned to the core epitope (defined in Figure 3C) of the reference structure (ZIKV sE/Fab C10 at 2.1-Å resolution, termed ZIKV sE^∗^), and the docking axis obtained after this alignment is displayed (Table S5). In the three panels, ZIKV sE^∗^ is shown in surface representation without the bound C10 Fab; only the C10 docking axis determined on this structure is shown in yellow, labeled “0.” A pink star to the right marks the location of the Asn67-linked glycan, present only on DENVs. Overlapped onto it are the docking axes determined for the 5 cryo-EM snapshots of (A) in the left panel, those determined for the 10 X-ray snapshots of sE/C10 complexes displayed in Figure 3A in the center panel, and the docking axes for Mab C8 in five available X-ray snapshots in the right panel. The docking axes in symmetric complexes are labeled 0 and the asymmetric ones 1 and 2 (and 1’ and 2′ when there are two dimers in the AU in the crystal) in the respective colors. 0 and 0’ in the third panel correspond to the X-ray structure of the ZIKV sE/C8 complex (PDB: 5LBS), which had two crystallographic half dimers in the AU. (C) Side view of C10^V^ and C8^V^ in the same orientation, with the CDRs highlighted. Note the bulkier projection of the C10 HCDR3.

**Figure S5:**
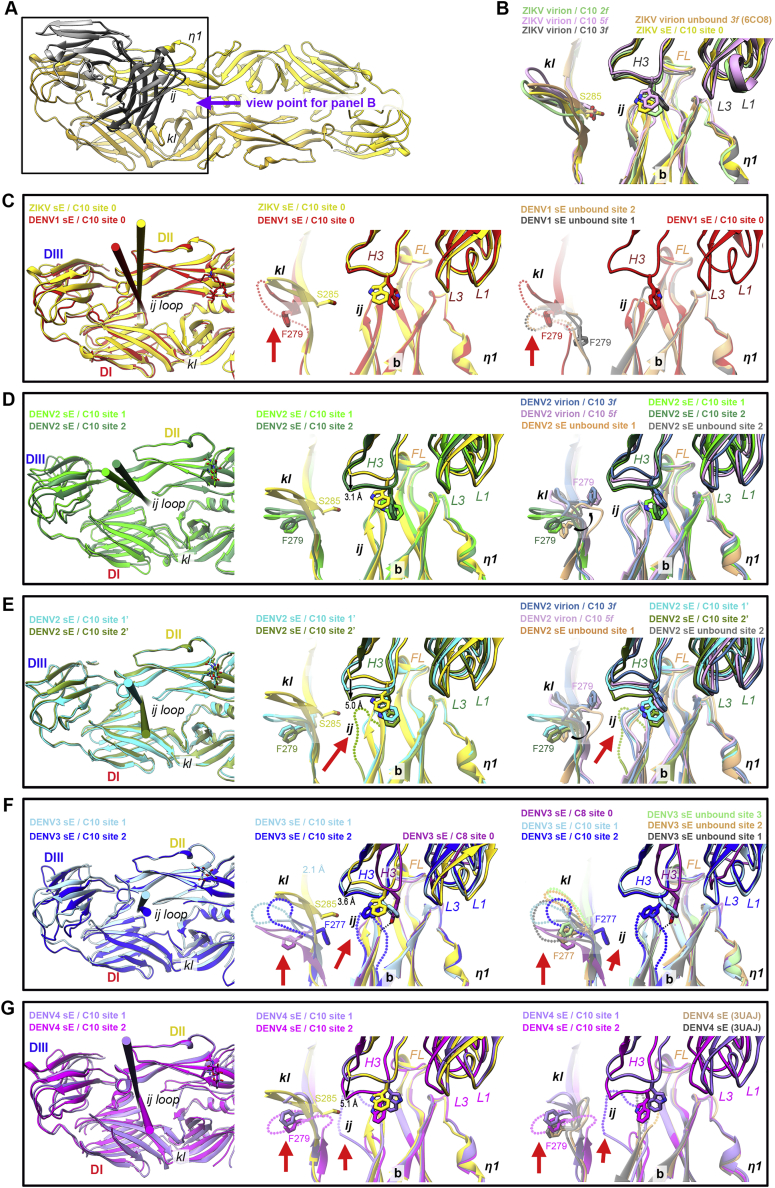
C10 HCDR3 interactions at the E dimer interface in the various structures, related to Figure 6 (A) The reference ZIKV sE/C10 Fab complex (Figure S2B, top panel; Table S2, first data column). For clarity, only one C10^V^ is displayed as gray ribbons, while ZIKV sE is in yellow. As explained in the text, for the comparisons in the other panels all the sE / C10 half-dimer snapshots were aligned on the core epitope at the tip of domain II, as defined in Figure 3C. (B) View down the purple arrow in (A) comparing the reference structure to the available cryo-EM snapshots of the ZIKV virion, C10 bound (PDB: 5H37, 4Å resolution) and unbound (PDB:6C08, 3.1 Å resolution). For clarity, both sE and C10^V^ of the reference structure are in yellow (here and in the middle panel of C-G below), and the various cryo-EM structures of the complex are displayed in a single color each, as indicated. The C10 paratope is presented with the HCDR3 (labeled as H3) projecting the prominent side chain of W^H100D^ as sticks, and also labeling the LCDR1 and LCDR3 loops as L1 and L3. The main elements composing the epitope are also labeled: fusion loop (FL), *ij* hairpin, *b* strand, *kl* hairpin across the E dimer interface and the *n*1 3/10 helical turn of the *bc* loop, which participates in I2/L2 interdimer contacts on the virion (see Figure 6). The side chain of Ser285 at the end of β-strand *l* (corresponding to Phe 279 in DENV1, 2 and 4, and Phe277 in DENV3, see alignment in Figure S2A) is highlighted as sticks, as this residue is referred to in the other panels. This panel shows that the sE protein in the X-ray structure adopts a conformation that matches that of E on virions, as the observed differences can be attributed to the lower resolution of the cryo-EM structures. This observation validates our choice of this particular structure as reference for all our comparisons. (C-G) Comparative analysis of the various DENVs structural snapshots reported to the reference structure, to unbound sE structures and to the cryo-EM structure of C10 bound to the virion in the case of DENV2. The left panels correspond to the boxed region in panel A. For clarity, C10^V^ is not shown, only the corresponding docking axes (extracted from the middle panel of Figure 5B). Sites 0, 1 and 2 are defined in the text and also in Figure S2, Figure S4C and S4). The middle panels show the same view as in panel B (down the arrow in (A), comparing in each case to the reference structure (yellow). The third panels compare the analyzed X-ray structures to various other structures available for E of the same virus, including the cryo-EM DENV2 / C10 structure presented here, and omitting the reference structure for clarity. Disordered regions are indicated in the middle and right panels by dotted lines of the same color as the ribbon diagram, with a thick red arrow highlighting them (C) DENV1 sE / C10 (Figure S2B, second panel; Table S2, second data column). The middle panel shows that the W^H100D^ side chain adopts a different rotamer than in the complex with ZIKV sE, and that the *ij* hairpins essentially overlap. The *kl* hairpin is partially disordered (dotted lines, marked by the red arrow). The DENV1 Phe279 and ZIKV Ser285 side chains are shown as sticks as a guide, as they mark the base of the *kl* hairpin at the end of the *l* strand (see the sequence alignment in Figure S2A). The right panel compares the structures of C10-bound and unbound DENV1 sE (determined here as it was not available in the PDB; it is displayed in Figure S2F; see also Table S2, seventh data column). The unbound structure is not symmetrical as the C10 bound sE, and the *kl* hairpin is disordered (red arrow), with density for Phe279 only on one half sE dimer (gray sticks). The *ij* hairpin is significantly shifted up with respect to the C10 bound form. This comparison indicates that sE in solution samples a broad conformational landscape, and that crystal packing of the unbound form selects for on asymmetric conformation. C10 binding appears, on the contrary, to select a symmetric conformation that matches better the conformation on virions (as the *ij* hairpin in the C10 bound DENV1 sE is closer to the reference structure, compare middle and right panels). (D) DENV2 sE / C10, complex 1 (from PDB:4UTC). The crystals used to determine this structure had two sE dimers bound to C10 in the asymmetric unit, arbitrarily termed here dimers 1 and 2; sE dimer1 makes the raft-like rows displayed in Figure 6E. The two sE half dimers are differentiated in two shades of green. Note that, compared to the reference structure, the HCDR3 projects deeper into the sE dimer interface (3.1Å, as labeled in the middle panel). This is not the case on virions, where the HCDR3 enters the sE dimer interface as in the reference structure (compare the H3 loop in the middle (yellow) and right panels (blue/pink, with *3f* site (in blue) being closest to the reference structure). Note also that on virions, Phe279 adopts a conformation closer to Ser285 in ZIKV (comparing with the middle panel) and points toward the E dimer interface. The curved black arrow in the right panel shows the transition of Phe279 from its location in the X-ray structures of sE (C10 bound and unbound) to that of E on virions. A similar change was observed in the first crystal structures reported for DENV2 sE (Modis et al., 2003), which showed a hydrophobic-ligand-binding pocket between the *kl* and *fg* hairpins (which are labeled in Figure 1A, bottom protomer) and in which the Phe279 side chain was found in different conformations depending on the presence or absence of the ligand. (E) DENV2 sE / C10, complex 2 (PDB:4UTC, second complex in the asymmetric unit), with the sE half dimers shown in light green and cyan (sites 1’ and 2′ respectively). The HCDR3 projects even deeper into sE dimer interface in site 2′, 5Å with respect to the reference structure (see middle panel), and the *ij* hairpin becomes disordered (red arrows). In site 1’, these elements remain similar to their counterpart in dimer 1 displayed in panel D. The Phe279 conformations also remain similar to those in dimer 1. (F) DENV3 sE / C10 complex (Figure S2B, third panel; Table S2, third data column). Different to the above panels, the middle panel shows, in addition to the two half sE dimers bound to C10 shown in dark blue and cyan, the structure of DENV3 sE bound to C8 (in purple/pink) (structure displayed in Figure S2E, see also Table S2, sixth data column). The *kl* hairpin is disordered in both DENV3 sE / C10 half dimers, but Phe277 (corresponding to Phe 279 in DENV2) is ordered in site 2 and oriented toward the sE dimer interface, although in a different conformation to Phe 279 on virions (compare the right panel with the panel immediately above). Compared to the DENV2 complexes, the C10 HCDR3 inserts at an angle, directed more toward the opposite sE subunit in the dimer (shifted by 3.6Å, labeled in the middle panel), and the side chain of W^H100D^ displays different rotamers in the two sites. The rotamer in site 2 interacts with a disordered *ij* hairpin (tilted red arrows). The DENV3 sE complex with C8 resulted in a symmetric, crystallographic dimer, in which both the *ij* and the *kl* hairpins were ordered. A crystal of unbound DENV3 sE had been reported to 3.7 Å resolution (Modis et al., 2005). Here we obtained crystals of unbound DENV3 sE which diffracted to 2.8Å resolution. The corresponding structure (Figure S2F; Table S2, eighth data column) showed an asymmetric sE dimer in which the *kl* hairpin was disordered (right panel). (G) the DENV4 sE / C10 complex (Figure S2B, fourth panel; Table S2, fourth data column. The half-dimer snapshots are shown in violet (site 1) and magenta (site 2). The HCDR3 loop inserts deep in site 2, pushing the *ij* hairpin toward the bottom (shown also in Figure S2D, inset) and a disordered *kl* hairpin. In the purple half-dimer, the *ij* loop is disordered despite a moderate insertion, similar to the ZIKV sE/ C10 complex (compared in the middle panel). There are no available structure of unbound DENV4 sE, so in the third panel we compare the DENV4 sE / C10 snapshots with the structure of DENV sE in complex with Mab 5H2 (Cockburn et al., 2012), whose epitope is away from the EDE. That structure was also not symmetrical, and so two half-sites are compared. A pattern similar to those described above is observed with respect to the kl hairpin and the orientation of Phe279.

Comparison of the cryo-EM structures in complex with the ZIKV and DENV2 virions indicated that C10 approaches the E protein in slightly different ways depending on the epitope location on the particle (*3f*, *2f*, and *5f*; Figure 5A), although the difference is smaller than in the case of the complexes with sE observed by X-ray crystallography (Figure 3A; compare the spread between domain III atoms at the opposite end of the dimer: 17.5 Å versus 37.6 Å) upon superposition of the C10^V^ backbone only (Table S4). The angle of approach in each case is seen better by comparing the docking axes as defined in Figure 5B (Table S5). Docking of C10 on the E dimer observed in the crystal structure of the ZIKV sE/C10 complex is essentially the same as that on the *3f* epitope on the ZIKV virion. On the DENV2 virion, only the docking axes at the *3f* and *5f* epitopes are visualized. They are tilted away from the glycan linked to Asn67 (pink star in Figure 5B), which is absent in ZIKV and influences the way C10 approaches the virus particle. This is highlighted by the docking axis on the ZIKV *5f* epitope, which shows that C10 adjusts to the contacts with the neighboring raft by tilting toward this position, which is forbidden by the presence of the glycan in the DENV particles (Figure 5B, left panel). Comparison with the X-ray structures of the sE/C10 complexes showed a subset of docking axes, most often only on one side of the sE dimer for the asymmetric structures, that remain similar to those seen on virions (Figure 5B, center panel). We found that the docking axes outside the oval represented in Figure 5B lead to clashes with the underlying E stem region and with protein M, as illustrated in Figures S2C and S2D. A further analysis of the X-ray structures of DENV2, DENV3, and DENV4 sE bound to C10 showed that its relatively long C10 HCDR3 loop wedges differently at the sE dimer interface in the three viruses, interacting with residues that are only partially accessible on the virion. In particular, the HCDR3 alters the conformation of the *ij* hairpin within the reference subunit and also that of the *kl* hairpin across the sE dimer interface (Figure S5). Both of these hairpins are affected in most of the snapshots. The C10 HCDR3 thus appears to knock on long-distance effects that alter the second epitope in the dimer. For further comparison, we obtained the X-ray structures of the DENV3 sE dimer in complex with Fab C8 (Figure S2E; Table S2), which targets the same epitope. Comparison with the available structures of ZIKV (PDB: 5LBS) and DENV2 (PDB: 4UTA) sE in complex with Mab C8 showed an undistorted, symmetric dimer with a narrow clustering of the C8 docking axes (Figure 5B, right panel). The differences in HCDR3 length of the two antibodies (Figure 5C) is likely responsible for the observed distortion when C10 binds E of DENV2, DENV3, and DENV4.

### Crystal packing mimics E dimer-dimer interactions on rafts

**Figure 6:**
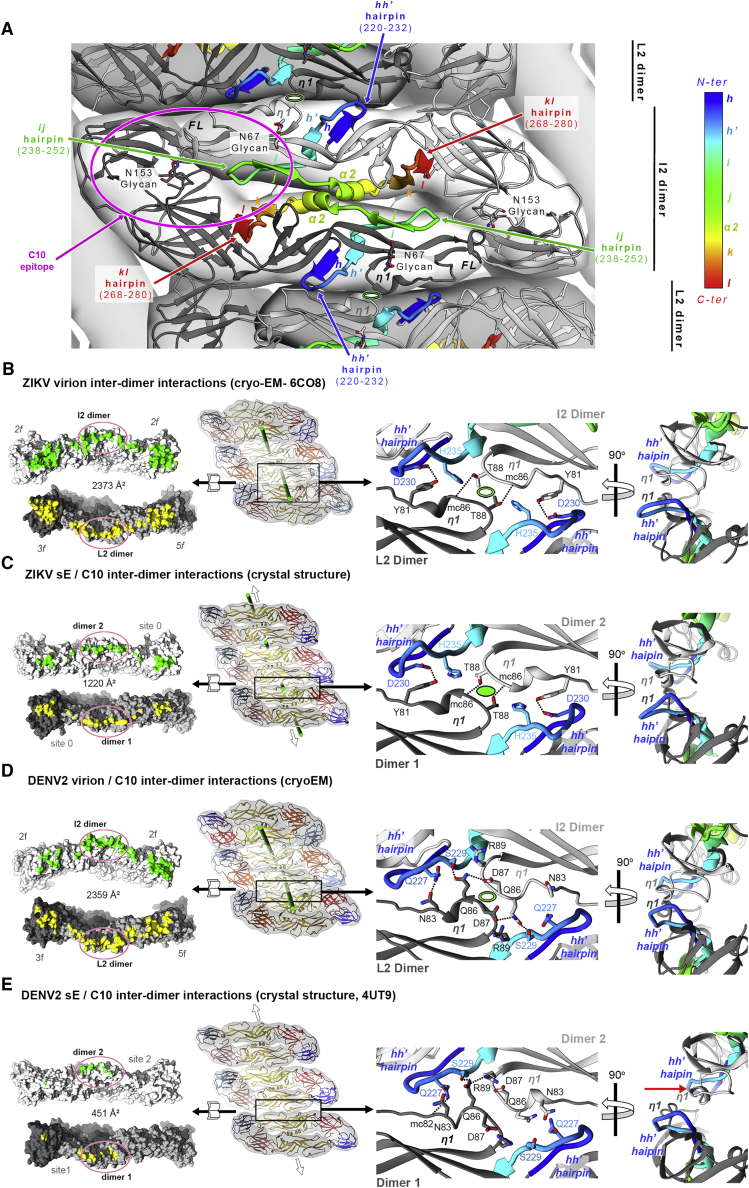
sE crystal packing mimics the lateral I2/L2 E dimer contacts on virions (A) View down an I2 axis of the 2.5-Å cryo-EM structure of the DENV2 virion (PDB: 7KV8) (Hardy et al., 2021). The segment with secondary structure elements *h*-*h’*-*i*-*j*-α2-*k*-*l*, is highlighted in a color ramp from the N to the C terminus (corresponding to aa 218–282 for DENV2 E; see Figure S2A, color key to the right). This segment is central to the E dimer and is involved in intradimer packing via the α2 helix and interdimer packing via the *hh’* hairpin, which contacts the *bc* loop of the adjacent E dimer via residues in the *n*1 turn (labeled). It is also involved in contacts with the underlying M protein via the *ij* hairpin and the α2 helix as well as in determining E dimer curvature via the *kl* hairpin. A magenta outline indicates the location of the C10 epitope to the left. (B–E) The *hh’*/ *bc* loop interdimer packing is preserved in the X-ray structures but is affected when both epitopes in the DENV2 sE dimer are bound by C10. The first column shows an open book view of the I2/L2 dimer contacts on the virion or between sE dimers in the crystals, with the BSA quoted. The second column shows the lateral packing of E dimers on virions or sE dimers in the crystals, as indicated, with a frame marking the enlarged area shown in the right panels. The quasi-2-fold (Q2) axes relating I2 and L2 dimers on virions, or crystallographic axes relating adjacent dimers in the crystals, are displayed in green. The third and fourth columns show a closeup of these contacts in two orthogonal views, highlighting the interaction between the *hh’* hairpin in one dimer with the *bc* loop (highlighted by labeling the η1 turn) of the adjacent dimer. The Q2 or strict 2-fold interdimer axis is marked by an empty or full oval, respectively. Hydrogen bonds across the dimer/dimer interface are shown as dotted lines. (B) L2 and I2 dimers on the Zika virion (PDB: 6CO8). (C) Adjacent dimers in the crystals of ZIKV sE/C10. Empty arrows indicate that the row of sE dimers extends indefinitely in the crystals. (D) Packing of I2 and L2 dimers in the C10-bound virion with roughly 50% occupancy, averaging E dimers with C10 bound at the *3f* or *5f* epitope. (E) sE dimer rows in theDENV2 sE/C10 crystals. In this structure, the dimers show propensity to pack in the same way as on the virion and on the crystals of the ZIKV/C10 complex (as in B–D), but the altered conformation of the sE dimer selected by C10 binding at both sites in solution results in a conformation in which the *hh’* hairpin faces the *bc* loop at one side only, losing the 2-fold symmetry of the contact (last panel, shift marked with an arrow).

The absence of C10 density at the *2f* epitopes on the DENV2 virions led us to more closely examine the contacts made by the central I2 dimer with its neighbors within a raft, in light of the asymmetric conformation observed in the X-ray structures. A central feature of the I2/L2 interdimer contacts is the interaction between the *hh’* hairpin with the long *bc* loop, which includes the *n1* 3/10 helical turn, of the opposite E dimer (Figure 6A). In the E protein, the *hh’* hairpin connects directly to the *ij* hairpin, which, in turn, is linked directly to the α2 helix, which mediates intra-dimer contacts. In turn, the α2 helix immediately precedes the *kl* hairpin, which controls the hinging of domain II with respect to domain I. Figure 6A displays the polypeptide segment in between *hh’* and *kl* hairpins in a color ramp from the N to the C terminus to highlight its central location in the E dimer and its position within the C10 epitope. This segment appears as a key element of the spring-loaded architecture of the E dimers in their metastable pre-fusion conformation on virions.

The buried surface area (BSA) at the contact between I2 and L2 E dimers in the virion is more than 2,000 Å^2^ per dimer and also involves inter-dimer contacts between domains I and III (red and blue domains, respectively, in Figure 1B). The extent of the buried surface suggests an intrinsic inter-dimer affinity, which is reflected in the packing in the ZIKV sE/Fab C10 crystals, which showed sE dimers packed laterally through the same interfaces as in the virion rafts. The BSA is smaller (1,200 Å^2^; Figures 6B and 6C), but the packing maintains the central *hh’*/*bc* loop contacts. With the sE dimers being less curved than E dimers on virions (Figures 6B and 6C, left two columns), the packing in the crystals resulted in straight rows of dimers extending indefinitely. In the DENV2 sE/C10 X-ray structure (PDB: 4UT9), one of the two complexes of the AU also displayed sE dimers packing via the same interface as I2 and L2 dimers on virions (Figure 6E), resulting in similar straight rows but with the notable difference that C10 binding introduced a distortion that abrogated the 2-fold symmetry of the contact and reduced the BSA to 450 Å^2^. The *bc* loop and the *hh’* hairpin still faced each other but were at different heights so that the *n*1 turn could not make the same interactions as on virions, resulting in looser contacts (compare Figures 6D and 6E). Although the docking axes of C10 on of the two epitopes of the DENV2/C10 complex (sites 1 and 2; Figures 5B, S4, and S5) remained in the allowed region (Figure 5B), the sE conformation adopted upon C10 binding at both epitopes interfered with formation of raft-like lateral contacts. Figure S2A shows that only in the case of DENV2 are residues of the *n*1 turn among those contacted by C10, suggesting that there is a specific rearrangement in this intra-raft contact region to adjust to the C10 paratope. This observed distortion is in line with the increase in hydrogen-deuterium exchange of DENV2 particles in peptides spanning this polypeptide segment upon C10 binding described by Lim et al. (2021). It is also in line with the observation of undetectable C10 binding to the I2 dimers on the virion, which have symmetrical *hh’* hairpin/*bc* loop contacts with L2 dimers at either side and are therefore less likely to sample asymmetrical E dimer conformations that can be selected by C10, unlike the L2 dimers.

### C10 IgG1 or F(ab’)_2_ cannot simultaneously bind both epitopes of the same sE dimer

**Figure S6:**
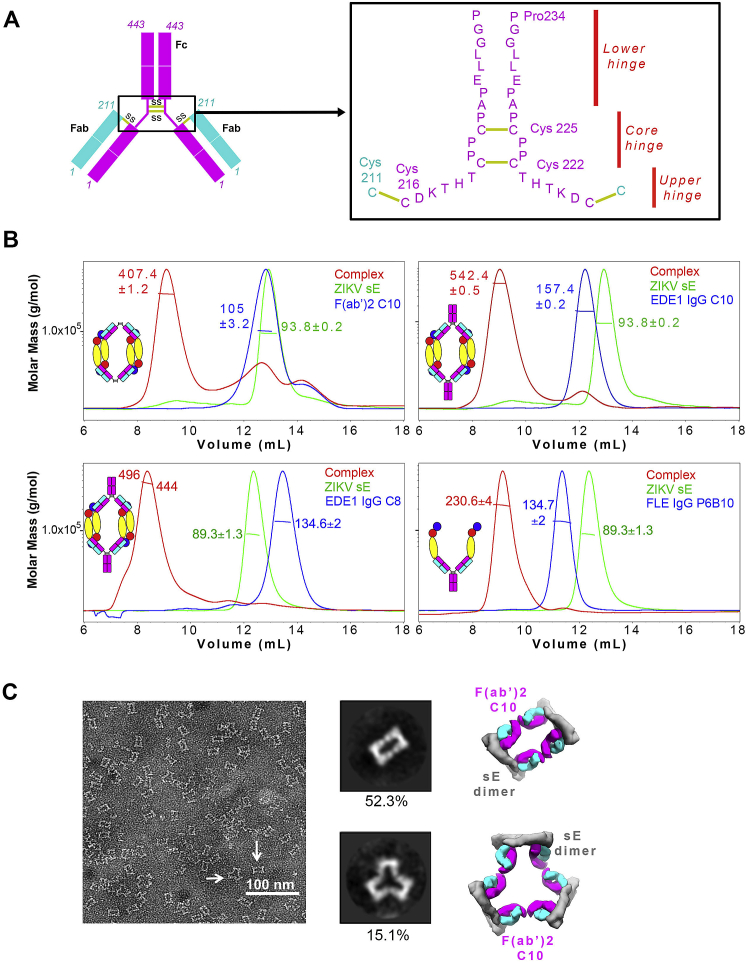
Bivalent C10 is unable to bind intradimer epitopes, related to Figure 7 (A) Diagram of a human IgG1 molecule (top) and sequence of the hinge linking Fab and Fc (inset, corresponding to the boxed area in the diagram). The heavy and light chains are represented in magenta and cyan, respectively. (B) SEC/MALS analysis of the isolated ZIKV sE, F(ab’)_2_ C10 alone and in complex with ZIKV sE (top-left panel); and of ZIKV sE, IgG C10 alone and in complex with ZIKV sE (top-right panel). The molecular weight determined by MALS is indicated, corresponding to the y axis on left. The cartoons illustrate the molecular complexes inferred from the molecular weights derived by MALS. The bottom panels show the SEC/MALS profile of ZIKV sE with IgG C8 (left panel) and with the FLE IgG P6B10 (right panel) alone and in complex. (C) Top, raw transmission electron micrograph of negative stained ZIKV sE / F(ab’)_2_ C10 complex. Middle panel, 2D class average of the complex with two major classes observed. Bottom, 3D cartoon representations of the two classes.

The immunoglobulins’ heavy chain CH1-CH2 hinge is 17-aa-long in human IgG1 and consists of a 4-residue core with the sequence ^H222^CPPC^H225^ (Kabat numbering), with the two cysteines making inter-heavy-chain disulfide bonds (Figure S6A). The IgG1 F(ab’)_2_ remains dimeric because it is truncated downstream of the CPPC core, whereas the Fab is truncated upstream. We studied the biophysical properties of the ZIKV sE dimer in complex with bivalent C10 IgG1 or F(ab’)_2_. Size-exclusion chromatography together with multi-angle light scattering (SEC-MALS) indicated that the complexes essentially had a stoichiometry of two IgG1 or F(ab’)_2_ C10 bound to two ZIKV sE dimers (2:2 stoichiometry; Figure S6B, top panel). We did not observe a peak corresponding to a 1:1 sE/F(ab’)_2_ complex, suggesting that C10 cannot bind bivalently to a single E dimer. We found the same behavior with mAb C8 (Figure 6B, bottom panel, left). As a control, we also monitored, by SEC-MALS, mAb P6B10, which targets the linear fusion loop epitope (FLE) (Dejnirattisai et al., 2015), in complex with ZIKV sE. The elution profile corresponded to a stoichiometry of one IgG1 for two sE monomers, confirming that, in solution, P6B10 dissociates the ZIKV sE dimer to access the fusion loop and binds an sE monomer per Fab arm (Figure S6B, bottom panel, right). Negative-stain EM analysis and 2D class averaging of the ZIKV sE/C10 F(ab’)_2_ complex showed images compatible with two sE dimers crosslinked by a F(ab’)_2_ molecule and, in some cases, three sE dimers with three F(ab’)_2_s (Figure S6C), corroborating that C10 cannot bind bivalently to a single sE dimer.

### The ZIKV sE / C10 F(ab’)_2_ crystal structure shows bivalent binding to the 3f epitope equivalents on a virion raft

**Figure S7:**
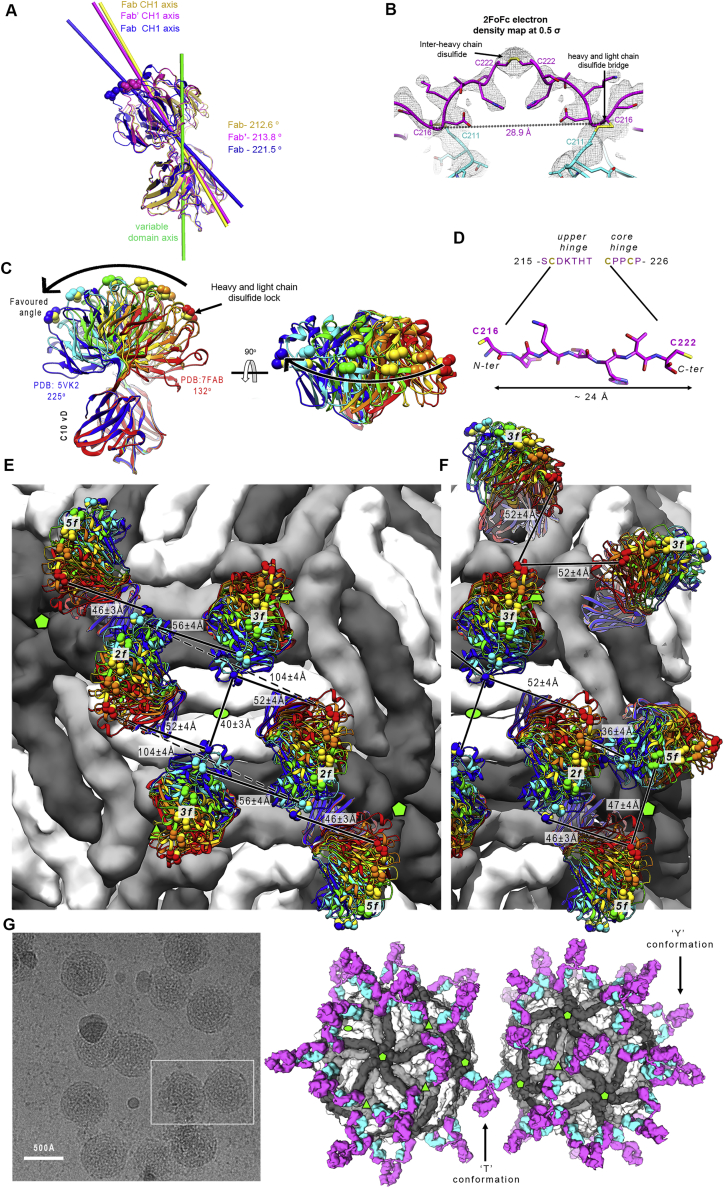
ZIKV sE in complex with bivalent C10, related to Figure 7 (A) Fab C10 elbow angle from the X-ray structures of the ZIKV sE in complex with Fab C10 (yellow), with F(ab’)_2_ C10 (magenta), and as modeled on the virion (blue). The Fabs were superposed on the variable domains (green axis). The angles were calculated using the online AS2TS server (Zemla et al., 2005). In the F(ab’)_2_ C10, the constant domain rotated by 1.2° toward its coupled arm, and a further 6.7° bending was required to connect the two Fab arms via an extended hinge when modeled on the *3f* epitopes. The elbow angle of 221.5° is within the accessible range, which spans from 132° to 225°, as shown in panel D (B) Electron density of the refined ZIKV sE / F(ab’)_2_ X-ray for the linker between the two Fab arms displayed at a low contour level (0.5 σ). The polypeptide chain was modeled only tentatively for representation purposes, as the density was very weak. This region is thus not present in the coordinates file deposited in the PDB. (C) Range of Fab elbow angles in selected structures from PDB database, ramp colored from red (smallest, 132°) to blue (largest, 225°) through yellow and green, and with the disulfide bond between C^L211^ and C^H216^ drawn as spheres. The PDB codes of the Fab structures with most extreme elbow angles are indicated. The C^H216^ Cα atom has an accessible range of about 50Å along the direction of the curved arrow (i.e, in the plane of the Figure). In our two crystal structures, the C10 Fab has a relatively large elbow angle (∼213 ^o^). (D) The IgG1 hinge segment extended to display the maximal distance between the C^H216^ and C^H222^ Cα atoms, which make disulfide bonds with the light chain C^L211^ in the Fab and with C^H222^ in the partner heavy chain of the antibody, respectively. This panel shows that the hinge between the two Fab arms of an IgG1 molecule, measured between the two C^H216^ Cα atoms can be stretched until ∼50 Å at most (i.e., the maximum distance is slightly longer than the distance of the fully stretched segment between the C^H216^ and C^H222^ Cα atoms in each heavy chain, allowing for the interchain disulfide bond). Although 50Å would then be the theoretical limit, this distance requires a fully stretched hinge segment, and can potentially be reached in the most favorable orientations of the two Fab arms with respect to each other. (E)-(F) These panels analyze potential bivalent binding within a raft (E) and across rafts (F). The combined flexibility about the Fab elbow angle and stretching of the heavy chain hinge allows bivalent IgG binding to only certain epitopes on virions. These panels show the antibody docked at the various epitopes with the whole range of elbow angles as shown in panel C. The shortest distances between the C^H216^ Cα atoms (using the adequate elbow angle, colored as in panel C) of adjacent Fabs are displayed. For instance, in panel E, dotted lines between Fab arms bound at the I2 dimer (the central white dimer) show that the closest distance is ∼100Å, demonstrating that these two epitopes cannot be bound by the Fab arms of the same IgG molecule, in line with the results in Figure S6. Although panel E shows Fab pairs different from those bound to the two *3f* epitopes spanning distances under 50Å, they involve in each case one *2f* epitope, which is bound poorly by C10, as shown by the cryo-EM reconstruction. Panel F suggests that inter-raft divalent binding to two adjacent *5f* epitopes could be feasible, as the closest distance is 47Å (bottom right). Yet contrary to the two Fab arms bound at the *3f* epitopes (shown in panel E at the center), in the inter-raft connection the Fabs cannot bend toward each other within the same vertical plane, but their mobility is about different planes and the linker requires some twisting to reach the second Fab, reducing the range it can extend. (G) Attempts to image of ZIKV virions with C10 F(ab’)_2_ by cryo-EM resulted in particle aggregation, as shown in the field view in the left panel. IgG1 C10 can bind bivalently to *3f* epitopes, and leaves the *5f* sites available for crosslinking (as outlined in the right panel) and precipitation of the sample. If the C10 F(ab’)_2_ could readily bind bivalently at the *5f* epitopes, the prediction would be that the virions would be less prone to aggregation.

**Figure 7:**
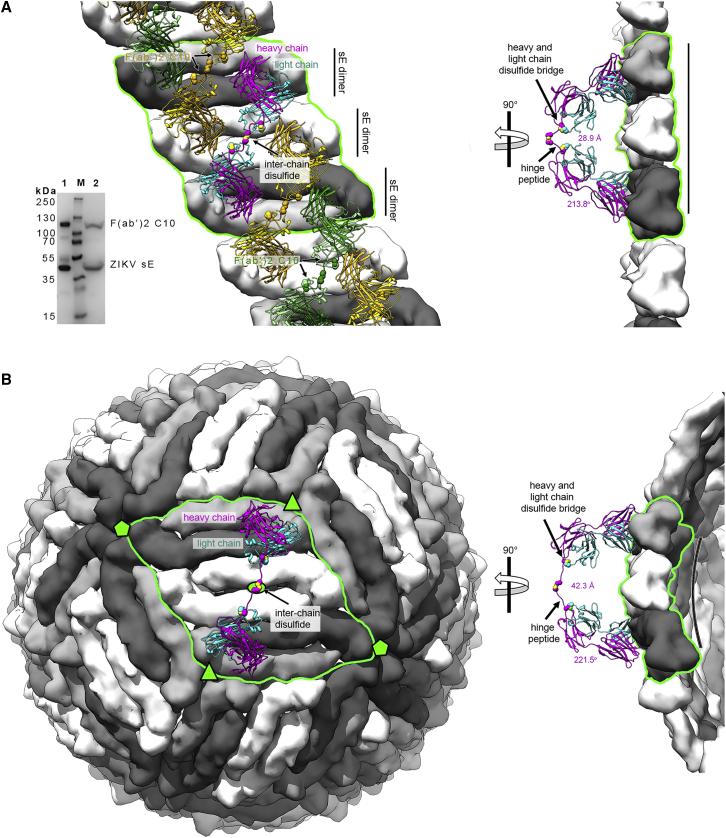
C10 binds bivalently at both *3f* epitopes per raft (A) Left: crystals of the ZIKV sE/C10 F(ab’)_2_ complex are formed of rows in which sE dimers pack laterally as in a raft (highlighted by a green contour). E dimers in the row are shown alternating white with light/dark gray dimers, as in Figures 1B and 4B. The cyan/magenta F(ab’)_2_ is related by the 2-fold molecular axis of a white dimer, and its Fab arms are bound to each of the two light/dark gray E dimers at either side. Similarly, the yellow F(ab’)_2_ shares the 2-fold molecular axis of a light/dark gray dimer and is bound to the two white dimers at either side, and so on. The hinge between the two Fab arms was modeled into weak density (Figure S7B). The panel on the right shows a side view of the row with only one C10 F(ab’)_2_ displayed for clarity. The distance between two heavy-chain C^H231^ Cα atoms and the elbow angle are indicated. The gel at the bottom left displays SDS-PAGE of the sE/F(ab’)_2_ complex isolated from a SEC column (lane 1) and from re-dissolved crystals (lane 2), demonstrating the presence of intact F(ab’)_2_ in the crystal. Lane M displays molecular weight markers (labeled at the left of the gel). (B) A F(ab’)_2_ C10 modeled on the Zika virion (PDB: 5H37) bound to the raft outlined in green. The right panel shows that the raft is curved, unlike the flat rows observed in the crystal shown directly above (in A). A change in the elbow angle brings the C-terminal ends of the C10 Fab heavy chain within reach of the CH1-CH2 hinge.

We obtained crystals of ZIKV sE in complex with C10 IgG and with F(ab’)_2_ that diffracted to 6-Å and 3-Å resolution, respectively, and determined the X-ray structure of the latter by molecular replacement (Table S2). The lateral packing between sE dimers was identical to that observed in the ZIKV sE/Fab complex (Figure S6B), but the Fab arms had a different elbow angle (Figure S7A). Although the antibody hinge region had weak density, it was possible to trace the ^H217^DKTHT^H221^ sequence of the upstream tether, which converged into a blob of density at the 2-fold axis that we assigned to the first inter-chain disulfide bond of the hinge core segment (Figure S7B). Because the density was weak, we re-dissolved the crystals and monitored them by non-reducing SDS-PAGE, which confirmed that they indeed contained an intact F(ab’)_2_ molecule (Figure 7A), ruling out any potential fortuitous cleavage in the linker region prior to crystal formation. Moreover, the two Fab arms connected by the weak density in the crystal were the only ones having the density for C^H216^ Cα atoms at the Fab C termini positioned within a distance range compatible with the hinge length (Figure S7D), supporting our interpretation of the bivalent binding mode.

The structure showed that the two arms of the C10 F(ab’)_2_ were bound to alternating sE dimers along raft-like rows (Figure 7A). When extrapolated to the virion, this result indicated that the two arms of bivalent IgG1 C10 can crosslink the two *3f* epitopes of each raft (Figure 7B). The curved surface on the virion compared with the flat E rows in the crystal would bring the C^H216^ Cα atoms of the two Fab arms farther apart, indicating that the hinge requires farther stretching than in the crystals, potentially requiring further Fab flexing about the elbow angle to keep the C10^V^ moiety unaltered on the epitope (compare the right panels of Figures 7A and 7B). Examination of the available Fab structures in the protein database indicated that the required adjustment is within the range of accessible elbow angles (Figures 7B, S7C, and S7D). This bivalent binding model indicates that 30 C10 IgG1s can completely coat a mature particle, one per raft (Figure 7C; Video S1), a number that has been shown to be sufficient to neutralize the particle (Pierson et al., 2007).

Video S1. Model of the binding of IgG1 C10 to ZIKV, related to Figure 7B

Analysis of the IgG1 hinge (Figure S6, Figure S7A, S7C, and S7D) shows that the distance between the C^H216^ Cα atoms from the two Fab arms within an IgG1 molecule must be under 50 Å. Considering the additional flexibility provided by the Fab elbow angles (Figure S7E), only the two *3f* epitopes can be bound bivalently because the other potential bivalent binding involve the *2f* epitopes (Figures S7E and S7F), which, as shown by our cryo-EM data, are not occupied. We conclude that binding to the two *3f* epitopes of each raft is the mode of binding and neutralization of IgG C10. The poor affinity of monovalent Fab binding suggests that the other epitopes will dissociate quickly and that only the antibodies bound bivalently at the *3f* epitopes will remain stably bound on DENV2, DENV3, and DENV4 particles. The fact that C10 IgG1 and F(ab’)_2_ neutralize DENV2 and DENV4 as strongly as ZIKV (Figure 1C) indicates that 30 C10 mAbs bound bivalently to each of the rafts are sufficient for neutralization. Full occupancy of the *3f* epitopes leaves the *5f* sites accessible to monovalent binding, in line with our attempts to make a cryo-EM reconstruction of DENV2 particles with C10 F(ab’)_2_, which resulted in precipitation of the sample, indicating that C10 can cross-link virus particles via the *5f* epitopes (Figure S7G).

## Discussion

### Conformational selection

Beyond the insight into the mechanism of cross-reactivity of C10, our analysis of the conformations of the E protein in solution and on virions in complex or not with C10, the details of which are provided in Figure S5, reveals key features to understand the intrinsic, functional conformational dynamics of E. These studies indicate that, in solution, sE displays a broad conformational landscape, sampling symmetric and asymmetric conformations. In the case of ZIKV and DENV1, the antibody can bind and stabilize a symmetric conformation of sE that is likely to be the most populated one. In the case of DENV2, DENV3, and DENV4, C10 binding selects for a presumably less populated asymmetric conformation, in line with the affinity studies (Figure 1C). Our analysis further indicates that such asymmetric states have altered intra-dimer contacts around the bound C10 HCDR3 loop. One affected element is the *kl* hairpin in the hinge between domains I and II of the opposite subunit in the dimer, which is known to have a long-range effect by altering the interactions at the distal tip (Zhang et al., 2004) located at the second epitope of the E dimer. Stochastically, then, the first binding event to one of the two epitopes results in a discreet change that allosterically affects the second epitope, which, we hypothesize, makes it even less optimal for binding. Further C10 binding to the second site selects for an sE conformation that appears to be accessible in solution but not on virions. With the I2 dimer being symmetrically constrained at the center of the virion raft, it does not sample the same conformational landscape as sE in solution and therefore cannot adopt a conformation that can be selected for C10 binding. Furthermore, C10 binding also affects the conformation of the I2/L2 interface on virion rafts in the case of DENV2 but not ZIKV (Figure 6). Similarly, the L2 dimer goes half of the way, sampling enough conformations so that C10 can select for the required asymmetric form, but in the environment on the virion, the second epitope cannot sample an even further asymmetric conformation so that C10 can bind, as in solution. As a result, we see C10 bound only with half occupancy to the L2 dimers (Figure S2C), and the predicted asymmetric state stabilized by C10 binding is not detected because of the icosahedral averaging to determine the structure. This asymmetric state of the DENV2 L2 dimer is expected to deviate less from a symmetric dimer than it does in the X-ray structures, in which both epitopes are occupied by C10.

### The *ij* hairpin as a potential key element of the “spring-loading” mechanism

Our extensive analysis of the allosteric effects of the C10 HCDR3 inserting at the dimer interface in the DENV2, DENV3, and DENV4 sE dimers (Figure S5) identified the *ij* hairpin as a potential sensitive element of a mechanism evolved by the virus to react to its environment and induce membrane fusion. The strictly conserved histidine in the *ij* hairpin (H249 in ZIKV; H244 in DENV1, DENV2, and DENV4; and H242 in DENV3; Figure S2A) has already been identified as essential for pH sensing (Kroschewski et al., 2009; Zheng et al., 2010). Here we made the unanticipated observation that the long HCDR3 loop of C10, which is nicely complementary to the C10 epitope in ZIKV and DENV1, in the other viruses is not. It instead selects an alternative conformation of the *ij* hairpin, which, in turn, affects the overall state of the E dimer and probably also its interactions on the virion because it appears to also affect the *hh’* hairpin at the L2/I2 dimer interface (Figure 6). The *kl* hairpin adopts variable conformations, being also often disordered in structures of unbound sE, which relate to the curvature of the sE dimers in each structure. In contrast, Figure S5 shows that the *ij* hairpin is affected only in the structures bound by C10. Attempts to force C10 binding to all its available epitopes per DENV2 particle by raising the temperature on a DENV2 strain that has been shown to change the surface packing of dimers with temperature (Fibriansah et al., 2013; Zhang et al., 2013) induces particle distortion, as described by Lim et al. (2021). These observations point to a potential effect of the C10 HCDR3 that partially releases the spring-loading mechanism by acting on the *ij* hairpin (Figure 6A). The very presence of C10 precludes the full fusogenic conformational change of E to proceed, as when triggered by acid pH. Lim et al. (2021) show that C10 indeed precludes particle aggregation by exposure of the fusion loops when treated at acid pH, but the aborted triggering of the conformational change leads to DENV2 particle distortion by altering the E dimer conformation.

### The orientation of the C10 docking axis allows bivalent binding to the 3*f* epitopes on virions

A number of studies have reported human antibodies cross-neutralizing ZIKV and DENV1, in particular antibodies targeting domain III (Keeffe et al., 2018; Oliphant et al., 2006; Robbiani et al., 2017; Rogers et al., 2017). The EDE antibodies are the only ones that expand cross-reactivity to include viruses of the other three DENV serotypes. Our results show that, affinity-wise, ZIKV and DENV1 indeed appear to be more closely related antigenically (Figures 1C and 2), despite the fact that bivalent C10 neutralizes DENV2 and DENV4 better than it does DENV1. However, C10 achieves its broader neutralization spectrum not via increased affinity for the more antigenically different viruses (Figure 1C). The geometry of the dengue virion makes that once bound to a *3f* site on a raft, and the second Fab arm is directed to make favorable interactions with the second *3f* site on the same raft, and such bivalently bound antibodies are likely to remain tightly bound, whereas monovalently bound C10 mAbs will be prone to rapid dissociation from DENV2 virions. Stretching the hinge and adapting the elbow angle of the Fab arms to accommodate the docking axes of the two Fab arms for bivalent binding thus has a major effect on antibody function, as demonstrated here for mAb C10. Because C8 shows similar docking axes (Figure 5B, right panel), the implications are that its expanded neutralization range is due to the same mechanism.

### Were the EDE antibodies originally elicited by ZIKV infection?

The EDE antibodies were cloned from plasmablasts of individuals in Vietnam who were hospitalized for dengue disease in November-December 2009. ZIKV became a global problem later, but there are a number of reports indicating that ZIKV had been circulating in South-East Asia for decades before reaching Polynesia and then jumping to South America (Ruchusatsawat et al., 2019; Wikan and Smith, 2017). Our results suggest that the individuals from whom the antibodies were isolated may have been primed by a ZIKV infection. Because in South-East Asia all four DENV serotypes circulate, it is possible that they had been further affinity matured for DENV2 and DENV4, given that they neutralize these two DENV serotypes as strongly as ZIKV. Although C10 affinity maturation is limited, we show here (Figure 2) that the light chain contains somatic mutations that are crucial for their cross-reactivity beyond ZIKV and DENV1.

Structural analyses of Fab antigen complexes, although highly informative, may underestimate the complexity of binding of bivalent antibodies to repetitive epitopes found on the surface of infectious pathogens. The structural rearrangements of Fab and E protein to accommodate bivalent binding and endow broadly neutralizing activity are remarkable. To develop an efficient vaccine eliciting broadly neutralizing antibodies against flaviviruses, it is not only complementarity to the epitope that is important but also the relative location of the epitopes targeted; i.e., the way in which they are presented at the immunogen’s surface. This study therefore raises interest in devising scaffolds to provide epitope presentation as similar to the mature viral particles as possible.

### Limitations of the study

Mixing bivalent C10 immunoglobulin with DENV2 virions under the conditions required for cryo-EM resulted in massive aggregation, precluding direct cryo-EM visualization of the bivalent binding observed in the crystals reported here. Lim et al. (2021), however, report a cryo-EM reconstruction of bivalent C10 F(ab’)_2_ bound as proposed here by capturing DENV2 virions on an EM grid and then adding more F(ab’)_2_ to further coat the particles. Although the resulting reconstruction did not directly visualize the IgG1 linker connecting the two Fab arms, the elbow angle of the bound Fab bent to match the predicted angle required for bivalent binding. Similar experiments using Fab instead of F(ab’)_2_ showed a very different, straight elbow angle, supporting the conclusion that bivalent binding to the 3*f* epitopes of each pair of L2 dimers per raft is what allows the very broad neutralization spectrum of Mab C10.

## STAR★Methods

### Key resources table

**Table:** 

REAGENT or RESOURCE	SOURCE	IDENTIFIER
**Antibodies**		

ALP-conjugated anti-human IgG	SIGMA	A9544, RRID:AB_258459
Goat anti-mouse IgG, conjugated with HRP	DAKO	P0447, RRID:AB_2617137

**Bacterial and virus strains**		

Dengue virus 1 strain Hawaii	Añez et al., 2016	GenBank: KM204119.1
Dengue virus 1 strain FGA/89	Duarte dos Santos et al., 2000	GenBank: AAF82039.2
Dengue virus 2 strain 16681	Goh et al., 2016	GenBank: KU725663.1
Dengue virus 2 strain NGC (cryo-EM)	Gruenberg et al., 1988	GenBank: AAC59275.1
Dengue virus 3 strain H87	Goh et al., 2016	GenBank: KU050695.1
Dengue virus 3 strain paH881	Unpublished NCBI/GenBank	GenBank: AAK18606.1
Dengue virus 4 strain 1-0093	Midgley et al., 2012	GenBank JQ740882
Dengue virus 4 strain Burma/63632/1976	Foster et al., 2003	GenBank: AAN38665.1
Zika virus strain PF13	Baronti et al., 2014	GenBank: MG827392.1

**Critical commercial assays**		

Platinum *Pfx* DNA Polymerase PCR mix	Invitrogen	11708-013
QIAquick PCR purification kit	QIAGEN	28104

**Deposited data**		

Coordinates and Structure Factors of DENV1 sE	This study	PDB: 7A3S
Coordinates and Structure Factors of DENV3 sE	This study	PDB: 7A3R
Coordinates and Structure Factors of DENV1 sE / scFv C10	This study	PDB: 7A3O
Coordinates and Structure Factors of DENV2 sE / scFv C10	PDB databank (Rouvinski et al., 2015)	PDB: 4UT9
Coordinates and Structure Factors of DENV3 sE / scFv C10	This study	PDB: 7A3P
Coordinates and Structure Factors of DENV4 sE / scFv C10	This study	PDB: 7A3Q
Coordinates and Structure Factors of ZIKV sE / Fab C10	This study	PDB: 7A3N
Coordinates and Structure Factors of ZIKV sE / F(ab’)2 C10	This study	PDB: 7A3U
Coordinates and Structure Factors of DENV3 sE / Fab C8	This study	PDB: 7A3T
Cryo-EM map of DENV2 virion / scFv C10	This study	EMBD: 30465
Coordinates of DENV2 virion / scFv C10 cryo-EM structure	This study	PDB: 7CTH

**Experimental models: Cell lines**		

D. melanogaster S2 cell line	Thermo-Fisher	Cat # R690-07
African green monkey kidney (Vero) cells	WHO reference cell bank	WHO Vero cells
HEK293T cells	ATCC	Cat # CRL-3216
C6/36 cells	AFRIMS	N/A

**Recombinant DNA**		

pMT/BiP/TwinStrep	Rouvinski et al., 2015	N/A
F(ab’)_2_ C10 pMT/BiP/TwinStrep	This study	N/A

**Software and algorithms**		

Coot 0.8.9.1	MRC	https://www2.mrc-lmb.cam.ac.uk/personal/pemsley/coot
UCSF Chimera 1.11.2	UCSF	https://www.cgl.ucsf.edu/chimera
UCSF ChimeraX 1.2.5	UCSF	https://www.rbvi.ucsf.edu/chimerax/
Phenix 1.14-3260	The PHENIX Industrial Consortium	https://phenix-online.org
MODELER	UCSF	https://salilab.org/modeller/
Pymol 1.7.2	Schrödinger	https://pymol.org/2/
XDS	Kabsch, 2010	https://xds.mr.mpg.de/
CCP4	Collaborative Computational Project, 1994	https://www.ccp4.ac.uk/
Phaser	McCoy et al., 2007	https://www.phaser.cimr.cam.ac.uk/index.php/Phaser_Crystallographic_Software
BUSTER-TNT	Blanc et al., 2004	https://www.globalphasing.com
Staraniso	Global Phasing Limited	https://staraniso.globalphasing.org/cgi-bin/staraniso.cgi
MolProbity	Williams et al., 2018	http://molprobity.biochem.duke.edu/
Prism	GraphPad	Version 7.0h
Astra 6	Wyatt Technology Corp	https://www.wyatt.com/products/software/astra.html
Relion 2.1	Scheres, 2012	https://www3.mrc-lmb.cam.ac.uk/relion/

### Resource availability

#### Lead contact

Further information and requests for resources and reagents should be directed to and will be fulfilled by the Lead Contact, Félix Rey (felix.rey@pasteur.fr).

#### Materials availability

Reagents generated in this study are available on request from the Lead Contact with a completed Materials Transfer Agreement.

### Experimental model and subject details

#### DENVs and ZIKV binding ELISA

To determine the binding affinity of mAbs to DENVs and ZIKV, DENV1 strain Hawaii (Añez et al., 2016), DENV2 strain 16681 (Goh et al., 2016), DENV3 strain H87 (Goh et al., 2016), DENV4 strain 1-0093 (Midgley et al., 2012), and ZIKV strain PF13 (Baronti et al., 2014) were produced in C6/36 cells were captured onto plate coated with 4G2 and then incubated with 1ug/ml of mAbs followed by ALP-conjugated anti-human IgG. The reaction was developed by the addition of PNPP substrate and stopped with NaOH. The absorbance was measured at 405 nm. Binding ratio was defined as the ratio binding or neutralization of mutant C10 with wild-type C10

#### Neutralization assays

The neutralization of mAbs was performed using Focus Reduction Neutralization Test (FRNT) as described previously (Dejnirattisai et al., 2015). Briefly, mAb was mixed with virus and incubated for 1 hr at 37°C. The mixtures were then transferred to vero cell monolayers and incubated for 2 days (for ZIKV) or 3 days (for DENV). The focus forming assay was then performed using anti-E Ab (4G2) followed by rabbit anti-mouse IgG, conjugated with HRP. The reaction was visualized by the addition of DAB substrate. The percentage of foci reduction was calculated for each antibody dilution. Neutralization ratio was defined as describe above.

#### EDE1 C10 mutation analysis

To compare the activity of EDE1 C10 antibody with germline sequence and contact residues of EDE1 C10 to epitope, heavy chain and light chain of EDE1 C10 were mutated by site-directed mutagenesis. EDE1 C10 were changed to germline sequence according to analysis from IMGT website (http://www.imgt.org) whereas the contact residues were converted to alanine. Briefly, EDE1 C10 expressing plasmids were subjected to Platinum® *Pfx* DNA Polymerase PCR mix (11708-013; Invitrogen) with specific primers which were designed by using QuikChange® Primer Design Program. PCR products were purified by QIAquick PCR purification kit (28104; QIAGEN) and treated with *DpnI* (R0176S; NEB) to remove parental plasmid DNA. PCR products were transformed into *E.coli*. All mutants were confirmed by sequencing. Plasmids were transfected into the 293T cell lines by Polyethylenimine method and culture supernatants were harvested for binding and neutralization assay.

#### Recombinant production of DENV and ZIKV sE proteins

DENV1-FGA/89 (aa 1-395) (Duarte dos Santos et al., 2000), DENV3-paH881 (aa 1-393), DENV4_Burma/63632/1976 (aa 1-395) (Foster et al., 2003) and ZIKV-H/PF/2013 (aa 1-404) sE proteins were cloned into a vector pMT/BIP/V5 with a tandem C-terminal strep-tag and expressed in *Drosophila* expression system (Invitrogen) as described previously (Barba-Spaeth et al., 2016; Rouvinski et al., 2015). Briefly, sE expression was induced by addition of 5 μM CuSO4 or CdCl2 and supernatants were harvested after 8–10 days post-induction. sE were purified using Streptactin columns (IBA) according to manufacturer’s instructions followed by size exclusion chromatography using Superdex 200 10/300 GL column equilibrated in 50 mM Tris (pH 8) and 500 mM NaCl. Disulfide engineered mutant dimers of DENV2 A259C (sE)_2_, DENV3 A257C (sE)_2_, DENV3 L107C/S313C (sE)_2_ and DENV4 A259C (sE)_2_ were produced as described earlier (Rouvinski et al., 2017).

#### Production of antigen binding (Fab) and single-chain variable fragment (scFv) of C10

C10 and C8 fragments were cloned into plasmids for expression of Fab and scFv in *Drosophila* S2 cells (Rouvinski et al., 2015). The constructs contain a tandem strep tag fused at the C terminus (only of the heavy chain in case of the Fab) for affinity purification. The purification protocol included a streptactin affinity column followed by gel filtration as described above. F(ab’)_2_ C10 construct was similar to Fab with the only difference that the construct contained extra IgG1 hinge peptide residues at the C terminus of the heavy chain. After streptactin purification, the dimeric F(ab’)_2_ was separated from the monomer fraction in gel filtration chromatography and the pure protein was used for assays.

##### Virus preparation (cryo-EM)

C6/36 cells were cultured at 32°C in the presence of 5% CO2. During cell passaging, we scraped cells from the dish, avoiding exposure of cells to trypsin. Thirty-five Corning tissue-culture treated culture dishes (D × H, 150 mm × 25 mm), each containing C6/36 cells in 30 mL of medium, were infected with DENV-2, New Guinea strain (Gruenberg et al., 1988). Four days after infection, cell culture medium was collected and was centrifuged for 30 min in a Beckmann centrifuge (11,000 g) to pellet large debris to be discarded. The supernatant harvested was centrifuged for 1 h in 26 × 38.5 mL centrifuge tubes in a Beckmann centrifuge (141,000 g) to collect the virus-containing pellet. The sample was left for 2 h at 4°C and subsequently the centrifuged virus was resuspended in PBS buffer by soaking of the pellet in the buffer for 10 min. The resuspended sample was then loaded at the top of a sucrose gradient (15% to 50%) and was centrifuged for 2 h at 130,000 g (Beckman Coulter SW41) at 4°C. A band was located at about one-third the distance from the top of the gradient. The gradient material above the band was removed with a pipette; then, the virus-containing band was carefully collected with another pipette, or the band was directly collected via a syringe. The collected viral sample (1 ml) was diluted to a volume of ∼12 mL with PBS buffer and an Amicon Ultra filter was used to concentrate the sample. The resulting 50 μL of purified virus was ready for cryo-EM.

### Method details

#### Real-time biolayer interferometry binding assays

Affinity of IgG1 C10 with purified DENV2 A259C (sE)_2_, DENV3 A257C (sE)_2_, DENV4 A259C (sE)_2_ and ZIKV (sE)_2_ (ZIKV sE is a natural stable dimer) proteins was accessed in real-time using a bio-layer interferometry Octet-Red384 device (Pall ForteBio). We didn’t use the WT DENV1, 2, 3 and 4 sE proteins because these are monomeric in solution and show monomer - dimer equilibrium, which adds to the measured signal and thus interferes with the experiment. We couldn’t produce DENV1 engineered cysteine sE dimer and thus they were not tested here. Anti-human IgG1 capture sensors (Pall ForteBio) were loaded for 10 min at 1,000 rpm shaking speed using IgG1 molecules at 10 μg ml^-1^ in assay buffer (25 mM Tris pH 8, 250 mM NaCl plus 0.2 mg ml-1 BSA and tween 0.01%). Unbound IgG1 molecules were washed away for 1 min in assay buffer. IgG1 loaded sensors were then incubated at 1,200 rpm in the absence and presence of two-fold serially diluted concentrations of DENV2-4 sE single cysteine mutant proteins and ZIKV sE for 15 min in assay buffer. Molar concentrations were calculated for the sE proteins in a dimeric form. For experiments with WT IgG1 C10, following range antigen concentrations were used: 2.3–300 nM (DENV2 sE dimer), 3.1-400 nM (DENV3 sE dimer) and 4.7-600 nM (DENV4 sE dimer) proteins and 2.3-300 nM (ZIKV sE dimer). For experiments with K66A mutant IgG1 C10, following antigen concentrations were used: 6.3–800 nM (DENV2 sE dimer), 3.1-400 nM (DENV3 sE dimer) and 37.5-4000 nM (DENV4 sE dimer) proteins and 2.3-300 nM (ZIKV sE dimer). Reference binding experiments were carried out in parallel on sensors loaded with control IgG1 mGO53 (Wardemann et al., 2003), which is a non DENV1-4 / ZIKV specific antibody. Operating temperature was maintained at 25°C. The real-time data were analyzed using Scrubber 2.0 (Biologic Software) and Biaevaluation 4.1 (GE Healthcare). Specific signals were obtained by double referencing, that is, subtracting non-specific signals measured on non-specific IgG1-loaded sensors and buffer signals on specific IgG1 loaded sensors. Association profiles, as well as steady-state signal versus concentration curves, were fitted assuming a 1:1 binding model.

##### Immune-complex formation and isolation

The purified antigens were mixed with Fabs or scFvs (in approximately 2-fold molar excess) in standard buffer (500 mM NaCl, 50 mM Tris pH 8). Samples were concentrated in Vivaspin 10 kDa cutoff to a volume of 0.5 ml. Post 30 min incubation in ice, the excess Fab/scFv C10 was separated from the antigen-Fab/scFv C10 complex by size exclusion chromatography in 50 Mm Tris (pH 8), 500 mM NaCl. In all cases, buffer was exchanged to 150 mM NaCl and 15 mM Tris pH 8 for crystallization trials. The protein concentrations used for crystallization, determined by measuring the absorbance at 280 mm, are listed in Table S2.

##### Crystallization and 3-D structure determination

Crystallization trials were performed in 400 nL sitting drops. The crystallization and cryo-cooling conditions are listed in the Table S2. Drops were formed by mixing equal volumes of the protein and reservoir solution in the format of 96 Greiner plates, using a Mosquito robot, and monitored by a Rock-Imager. Crystals were optimized with a robotized Matrix Maker and Mosquito setups on 400 nL sitting drops or manually in 24-well plates using 2-3 μL hanging drops. The crystals obtained were tested at several beam lines at different synchrotrons: SOLEIL (St Aubin, France) and ESRF (Grenoble, France). The datasets were indexed, integrated, scaled and merged using XDS (Kabsch, 2010) and AIMLESS (Evans and Murshudov, 2013). The structures were then determined by molecular replacement with PHASER (McCoy et al., 2007) using the search models listed in Table S2. Because of the anisotropy of the DENV3 sE / C8 Fab crystals, the DEBYE and STARANISO programs developed by Global Phasing Ltd. were applied to the AIMLESS scaled data without truncation of the resolution, using the STARANISO server (https://staraniso.globalphasing.org/). These programs perform an anisotropic cut-off of merged intensity data with a Bayesian estimation of the structure amplitudes, and apply an anisotropic correction to the data. These corrected anisotropic amplitudes were then used for further refinement of that structure with PHENIX (Terwilliger et al., 2009; Table S2). In general, all the models were alternatively manually corrected and completed using COOT (Emsley and Cowtan, 2004) and refined using either PHENIX or BUSTER-TNT (Blanc et al., 2004). For some structures, the refinements were constrained using non-crystallographic symmetry (Table S2).

#### SEC-MALS analysis and non-reducing SDS-PAGE

SEC-MALS was performed by loading ∼150 ug of ZIKA sE, F(ab)’2 C10, IgG1 C10, IgG1 C8 and IgG1 FLE P6B10 protein into Superdex 200 10/300 GL column (GE life sciences). Samples were run in Tris 50 mM pH 8.0 and NaCl 500 mM at a flow rate of 0.4 ml min^−1^. Sample molecular weight was detected by a Wyatt DAWN Heleos II EOS 18-angle laser photometer coupled to a Wyatt Optilab TrEX differential refractive index detector. Data were later analyzed using Astra 6 software (Wyatt Technology Corp). ZIKV sE / F(ab)’2 C10 complex crystals (∼20 crystals) were fished from the manual drops and serially washed in two drops containing the crystallization solution by transferring crystals to these drops. These washed crystals were loaded with non-reducing SDS-PAGE dye.

#### Negative stain electron microscopy and data analysis

Purified ZV sE / F(ab’)_2_ C10 complex was negatively stained with 1% uranyl acetate on grids coated with carbon film. Six micrographs were then manually recorded on a 4k × 4k CCD camera at 70,000 × magnification with a 10 s pre-exposure in an FEI Tecnai F20 electron microscope operated at 200 kV. For 2D image analysis, 1043 particles were picked from the micrographs and reference-free 2D classification were performed with Relion (Scheres, 2012) to generate 2D class averages.

#### Cryo-EM reconstruction

##### Electron microscopy

Purified virus was mixed with scFv C10 (1:4, vol/vol). The final scFv concentration was 80 μM, ensuring a molar excess to saturate the epitopes on virions. Aliquots of 3 μL of the mixture were placed on glow-discharged holey carbon grids (Quantifoil Cu R2/2), glow-discharged was performed via Gatan Solarus Plasma Cleaner (H_2_&O_2_, 12 s). Grids were blotted for 5 s under 20°C/100% humidity with blot force 4 and were flash frozen in liquid ethane with an FEI Vitrobot Mark IV. Grids were transferred to an FEI Titan Krios electron microscope operating at 300 kV. Images were directly recorded by Leginon with a Gatan K2 Summit detector without energy filter in counting mode on the Titan Krios microscope at a nominal magnification of × 29,000 (which yields a pixel size of 1.28Å), only one image was taken in one hole after one stage shift. Underfocus values in the final K2 dataset ranged from 0.2 μm to 3.5 μm, and 25 frames of each movie were used for later image processing.

##### Image processing

Particles were picked with Ethan (Kivioja et al., 2000), 2D classification of particle images was performed with relion, good particles from 2D class averages with distinct transmembrane density across the lipid bilayer of the virion were selected for futher processing. We selected more than 10,000 particles with a box size of 640 pixels from 1,035 micrographs in the final K2 dataset. Contrast transfer function parameters were estimated with CTFFIND4 program for finding contrast transfer functions in electron micrographs. We used cisTEM’s Ab-Initio 3D Reconstruction action to generate a starting model, then we used cisTEM’s 3D Auto Refinement action to get the final map, after one round of defocus & beam tilt refinement. The final resolution of the map reached 3.28Å.

DENV2 envelope and M protein from PDB 7KV8 (Hardy et al., 2021) were used along with scFv C10 PDB (from our crystal structure reported here- PDB 7A3N) for initial fitting into the map using chimera. Residues different in DENV2 PDB 7KV8 from our DENV2 strain used here were mutated using coot. Fitted structures were refined using real-space refinement in Phenix (Terwilliger et al., 2009). Ramachandran restrains, secondary structure restrains, NCS and a weight of 2.5 was applied during the refinement. Quality of refined models after each cycle were assessed using validation reports generated after refinement. Our final refined model was with 0.05% Ramachandran outliers, 0.44% rotamer outliers, 0.0 Cβ outliers, 12.14 Clash score and a MolProbity (Williams et al., 2018) score of 1.79 (please see full validation report for PDB 7CTH for more parameters).

### Quantification and statistical analysis

#### Analysis of the atomic models and illustrations

Each complex was analyzed with the CCP4 suite of programs. For intermolecular interactions, the maximal cut-off distance used for the interactions was 4.0 and 4.75 Å for polar and van der Waals contacts, respectively. Multiple sequence alignments were calculated using Clustal W and Clustal C version 2 on the EBI server. The figures were prepared using ESPript (Robert and Gouet, 2014), pyMOL Molecular Graphic System, version 1.8.2.0 (Schrödinger) (http://pymol.sourceforge.net) and UCSF Chimera version 1.11 (Pettersen et al., 2004). The Ab sequences were analyzed by Abysis (http://www.abysis.org/abysis/) (Swindells et al., 2017) and IMGT (http://www.imgt.org/) (Lefranc et al., 2009) websites for mapping CDR/framework regions according to Kabat and IMGT. RMSD calculations for Figure 3B were performed using ProSMART (Nicholls et al., 2014). C10 docking axes and epitope RMSD were calculated using both PyMOL (The PyMOL Molecular Graphics System, Version 2.0 Schrödinger, LLC.) and UCSF Chimera (Pettersen et al., 2004). The C10 Docking axes were calculated for the beta- barrel part of C10 variable domains for each complex.

#### scFv C10 Occupancy calculation

The occupancy was measured using a home-made UCSF chimera plugin called MDen, as described earlier (Liu et al., 2017). Briefly, the relative density ratio between symmetry related scFv C10 molecules (*3f* and *5f*) and the asymmetric unit of DENV2 envelope protein was calculated. The densities of scFv C10 CDRs and E asymmetric unit were first segmented in the unsharpened EM map using UCSF Chimera (Pettersen et al., 2004). CDRs are defined as residues 26-32, 49-53, and 89-97 (in Kabat numbering) for the light chain, and residues 25-33, 51-57, and 93-102 (Kabat numbering) for the heavy chain. A mask was generated via Relion’s relion_mask_create command aiming for reaching a unified ratio (here 4.2 in this study) between the calculated molecular weight of scFv CDRs or E asymmetric unit (calculated using the ExPASy server- (Gasteiger et al., 2003)) versus their non-negative density value sum. A relative density value was then calculated using the generated mask and the unsharpened EM map via MDen for each density parts (scFv at *3f*, *5f* and E asymmetric unit). To estimate the occupancy, scFv C10 values at *3f* and *5f* were divided by the E protein asymmetric unit value to get the final ratios of ∼55% and 45% at *3f* and *5f*, respectively.

## Data Availability

Coordinates, structure factor files and cryo-EM maps are deposited in the Protein Data bank and Electron Microscopy Data Bank with PDB: 7A3N, 7A3O, 7A3P, 7A3Q, 7A3R, 7A3S, 7A3T, 7A3U, 7CTH, and EMDB: 30465. Any additional information required to reanalyze the data reported in this work paper is available from the lead contact upon request.

## Supplemental information

Table S1. Results for binding and neutralization of the EDE1 C10 germline and alanine scanning of contact residues on ZIKV and DENV1–DENV4, related to Figure 2

Table S2. Crystallization conditions, diffraction data collection and refinement statistics, cryo-EM conditions, and data processing model statistics, related to Figures 3A and 4A

Table S3. List of all polar and electrostatic interactions observed in the crystal structures of C10 in complex with sE from ZIKV and DENV1-4, related to Figure 3A

Table S4. RMSD (Å) of the aligned structures, related to Figures 5A and 5B

Table S5. C10 docking axes, *ij* hairpin conformation, and BSAs, related to Figure 5B
