# Supplementary material for: The epitope arrangement on flavivirus particles contributes to Mab C10’s extraordinary neutralization breadth across Zika and dengue viruses
Source: Cell. 2021 Dec 9;184(25):6052–6066.e18. doi: 10.1016/j.cell.2021.11.010 (PMC8724787; doi:10.1016/j.cell.2021.11.010)
Supplement: Table S4. RMSD (Å) of the aligned structures, related to Figures 5A and 5B [file mmc4.pdf]

Table S4. RMSD (Å) of the aligned structures in Fig. 5A-B.

Related to Fig. 5A.

RMSD for C10 vD Cα atoms (Å)	**DENV2 virion / C10 *5f***	**ZIKV virion / C10 *2f***	**ZIKV virion / C10 *3f***	**ZIKV virion / C10 *5f***
**DENV2 virion / C10 *3f*** (reference structure)	0.64	0.86	0.97	0.90

Related to Fig. 5B

RMSD for the DII tip (Å)	**DENV2 virion / C10 *3f***	**DENV2 virion / C10 *5f***	**ZIKV virion / C10 *2f***	**ZIKV virion / C10 *3f***	**ZIKV virion / C10 *5f***	**DENV1 sE / scFv C10 site 0**	**DENV2 sE / scFv C10 site 1**	**DENV2 sE / scFv C10 site 2**	**DENV2 sE / scFv C10 site 1'**	**DENV2 sE / scFv C10 site 2'**	**DENV3 sE / scFv C10 site 1**	**DENV3 sE / scFv C10 site 2**	**DENV4 sE / scFv C10 site 1**	**DENV4 sE / scFv C10 site 2**	**DENV2 sE / Fab C8 site 1 (4UTA)**	**DENV2 sE / Fab C8 site 2 (4UTA)**	**DENV3 sE L107C-A313C / Fab C8 site 0**	**ZIKV sE / scFv C8 site 0**	**ZIKV sE / scFv C8 site 0'**
ZIKV sE / Fab C10 site 0 (reference structure)	0.64	0.83	0.60	1.05	1.12	0.34	0.70	0.72	0.60	0.79	0.56	0.64	0.47	0.77	0.49	0.53	0.61	0.42	0.43

For C10 vD, RMSD was calculated in comparison to the DENV2 virion C10 *3f* reference structure. All C10 vD are aligned on to residues 3-109 of heavy chain and 2-102 of the light chain (Kabat numbering), and RMSD for the 214 Cα pairs is reported in each column.

Strutures aligned onto the DII tip of ZV s / Fab C10 struture (residues 6-120). RMSD for the 55 Cα atom pairs in each column. Columns with orange background are related to the left panel in Fig. 5B, columns with green background are related to the middle panel and columns with blue background are related to the right
