# Supplementary material for: The epitope arrangement on flavivirus particles contributes to Mab C10’s extraordinary neutralization breadth across Zika and dengue viruses
Source: Cell. 2021 Dec 9;184(25):6052–6066.e18. doi: 10.1016/j.cell.2021.11.010 (PMC8724787; doi:10.1016/j.cell.2021.11.010)
Supplement: Table S5. C10 docking axes, ij hairpin conformation, and BSAs, related to Figure 5B [file mmc5.pdf]

Table S5. C10 docking axis, *ij* hairpin conformation and the buried surface area (Related to Fig. 5B and S5)

Structure	Docking axis (°)#	*ij* loop conformation	Buried Surface Area (Å^2)		
			Reference subunit	*Opposite subunit*	*N67 Glycan*
ZIKV sE / C10 site 0	0.0	ordered	666,1	607,8	0
DENV1 sE / C10 site 0	3,85	ordered	752,5	535,6	103
DENV2 sE /C10 site 1	9,02	ordered	690,5	352,5	76,4
DENV2 sE /C10 site 2	7,82	ordered	690,8	360,2	70
DENV2 sE /C10 site 1'	7,38	ordered	704,7	368,1	82,8
DENV2 sE /C10 site 2'	14,79	disordered	643,8	445,1	70,4
DENV3 sE /C10 site 1	7,81	ordered	713,2	385,6	83,1
DENV3 sE /C10 site 2	10,28	disordered	574,4	530,7	0
DENV4 sE /C10 site 1	3,58	disordered	589,3	379,6	53,5
DENV4 sE /C10 site 2	13,06	disallowed (see fig. S2G)	637,7	440,1	62,4
DENV2 virion / C10 *3f*	3,35	ordered			
DENV2 virion / C10 *5f*	5,51	ordered			
ZIKV virion / C10 *2f*	1,26	ordered			
ZIKV virion / C10 *3f*	2,13	ordered			
ZIKV virion / C10 *5f*	4,83	ordered			

Orientation with respect to C10 bound to ZIKV sE (Site 0)

ZIKV virion / C10 PDB code is 5H37

For the defination of Refernce and Opposite subunits in the BSA (Buried Surface Area) columns, please refer to Fig. 1A
